# Association between T cell exhaustion and the recurrence of atrial fibrillation after cryoballoon ablation

**DOI:** 10.3389/fimmu.2026.1767253

**Published:** 2026-04-14

**Authors:** Rong Suo, Shiyu Qi, Wenhua Song, Bingshuo Shi, Haipeng Zhang, Daiqi Liu, Tong Liu, Xing Liu

**Affiliations:** 1 Tianjin Key Laboratory of Ionic-Molecular Function of Cardiovascular Disease, Department of Cardiology, Tianjin Institute of Cardiology, The Second Hospital of Tianjin Medical University, Tianjin, China; 2 Department of Cardiology, Tianjin Hospital, Tianjin, China; 3 Department of Clinical Laboratory, The Second Hospital of Tianjin Medical University, Tianjin, China

**Keywords:** atrial fibrillation recurrence, cryoballoon ablation, exhausted t cell, immunity, immunosenescence, KLRG1, PD-1

## Abstract

**Background:**

Atrial fibrillation (AF) recurrence remains a challenge after Cryoballoon ablation (CBA). To date, few studies have investigated the impact of immunosenescence, particularly exhausted T cells, on AF recurrence after CBA. This study aims to investigate the association between exhausted T cells and AF recurrence after CBA, and to develop a novel model for the prediction of AF recurrence.

**Methods:**

Clinical data of patients undergoing CBA treatment for AF at Tianjin Medical University Second Hospital from March to November 2022 were collected, and AF recurrence was followed up. Flow cytometry was employed to evaluate the levels of inhibitory receptors (IR) on exhausted T cells, including killer cell lectin-like receptor G1 (KLRG1), programmed death-1 (PD-1), T cell immunoglobulin and mucin domain-containing molecule 3 (Tim-3), lymphocyte activation gene 3 (LAG-3), CD27, and CD57. Variables selected through Lasso regression analyses were incorporated into a multivariable Cox proportional hazards model to validate and identify independent risk factors for AF recurrence, building two predictive models. The efficacy of the two models was assessed using Receiver Operating Characteristic (ROC) curves, calibration curves, and Decision Curve Analysis (DCA).

**Results:**

Cox multivariate regression analysis revealed that KLRG1 of CD8^+^ T central memory cell (Tcm), PD-1 of CD8^+^ naive T cell (Tnaive), mean corpuscular volume (MCV), γ-Glutamyl Transferase (γ-GGT), glucose, and mitral valve maximum blood flow velocity (MV Vmax) were significant predictors. Two models were developed based on Cox multivariate regression analysis. Model 1 includes MCV, γ-GGT, glucose, and MV Vmax, while Model 2 includes all risk factors: MCV, γ-GGT, glucose, MV Vmax, KLRG1 of CD8^+^ Tcm, and PD-1 of CD8^+^ Tnaive. Validation of both models revealed that Model 2 showed better predictive performance, and a nomogram was created to present the results visually.

**Conclusion:**

The KLRG1 of CD8^+^ Tcm and PD-1 of CD8^+^ Tnaive are novel independent risk factors for AF recurrence after CBA and play a significant role in the early recurrence of AF. We constructed a new predictive nomogram incorporating KLRG1 of CD8^+^ Tcm and PD-1 of CD8^+^ Tnaive as key variables, which can enhance the predictive value for AF recurrence in patients after CBA surgery.

## 1 Introduction

Atrial fibrillation (AF) is the most common form of cardiac arrhythmia encountered in clinical practice, associated with substantial morbidity and mortality due to a range of potentially serious complications, including cardiomyopathy, heart failure, and stroke (1). With the increasing global population aging, it is projected that by 2050, the number of AF patients in the United States will reach 6 to 12 million, and by 2060, this number will rise to 17.9 million in Europe (2). The trigger and reentry mechanisms are typically located at the junction between the pulmonary veins and the left atrium (3). For patients with AF, there are several treatment methods, with Cryoballoon ablation (CBA) aimed at pulmonary vein isolation being a safe and effective option (4). However, the recurrence rate of AF after this procedure remains as high as 25%–30%, leading to an increased incidence of complications and the need for repeat surgeries (5). Therefore, it is crucial to improve the prognosis of post-CBA AF patients by accurately identifying and timely intervening in high-risk populations for recurrence.

T cell exhaustion is defined as the development of a dysfunctional phenotype in T cells caused by prolonged antigen exposure in chronic infections and tumor environments, leading to diminished effector function and reduced ability to eliminate target cells (6). Exhausted T cells lose robust effector functions, express multiple inhibitory receptors (IR) (including programmed cell death protein 1 (PD-1), T-cell immunoglobulin and mucin-domain containing-3 (TIM-3), lymphocyte activation gene 3 (LAG-3), and killer cell lectin-like receptor G1 (KLRG1)), and are characterized by an altered transcriptional program (7). It has been extensively studied in the context of tumors and immune system diseases (8).

As an IR of the C-type lectin family, KLRG1 exerts its immunosuppressive effects by binding to its ligands E-cadherin, N-cadherin, and R-cadherin. This interaction recruits the phosphatases SHIP-1 and SHP-2, which mediate inhibitory signaling, thereby downregulating the activity of the PI3K/Akt pathway. This cascade leads to reduced T-cell proliferation, decreased cytokine secretion, and ultimately induces cellular senescence or exhaustion (9–11). In diseases such as chronic lymphocytic leukemia, chronic hepatitis B, and colorectal cancer, the KLRG1-positive T-cell subset is significantly expanded, and its expression level is closely associated with disease progression, therapeutic response, and prognosis (12–14). Furthermore, KLRG1 is co-expressed with other IRs, including PD-1, 2B4, and CD160, forming a synergistic inhibitory network that exacerbates the T-cell exhaustion phenotype (15, 16). As a key marker of exhausted T cells, KLRG1 has been validated as an effective discriminator between functionally impaired and normally functional T-cell subsets (17).

As a member of the immunoglobulin superfamily, PD-1 is a membrane protein consisting of 288 amino acids and a key immune checkpoint molecule during T cell exhaustion (18). PD-1 was originally cloned from apoptotic murine T cell hybridomas. Upon binding to its ligands, PD-1 mediates cell cycle arrest rather than directly inducing cell death (19). In immune cells, PD-1 is predominantly expressed on the surface of activated T cells, B cells, natural killer cells, monocytes, and dendritic cells, with expression initiated during the early stage of T cell priming (20). The ligands of PD-1 comprise PD-L1 and PD-L2. PD-L1 exhibits a broader expression profile, detectable not only on immune cells but also on various cell types, including tumor cells, vascular endothelial cells, and mesenchymal stem cells. In contrast, PD-L2 is mainly expressed on macrophages and dendritic cells (21). Engagement of PD-1 with its ligands ultimately results in suppressed T cell proliferation, reduced cytokine secretion, and impaired effector functions (22). Within the tumor microenvironment, the PD-1/PD-L1 pathway has been established as one of the critical mechanisms underlying tumor immune escape (23). Moreover, sustained activation of this pathway is central to T cell exhaustion and compromised immune function in pathological conditions such as chronic infection (24).

In recent years, with the intersection and integration of immunometabolism and cardiovascular pathophysiology, the role of Exhausted T cells in cardiovascular diseases (such as acute myocardial infarction (25), acute coronary syndrome (26), etc.) has gradually become a research hotspot. AF, a chronic inflammatory disease, is characterized by a persistent inflammatory microenvironment and dysregulated immune cell function during its pathogenesis (27). Additionally, the tissue repair process following CBA may trigger aberrant local immune responses. These pathophysiological features collectively provide the necessary conditions for the development of T-cell exhaustion. However, to date, no studies have thoroughly investigated the impact of exhausted T cells on the recurrence of AF post-CBA.

Based on follow-up data from patients with post-CBA AF, this study collected relevant clinical and laboratory data to systematically explore the relationship between exhausted T-cell markers, particularly KLRG1 and PD-1, and AF recurrence following CBA. Furthermore, we integrated these findings with clinical characteristics to establish a scientific risk prediction model. This study aims to provide a novel theoretical basis and potential therapeutic targets for the precise identification of high-risk patients and the implementation of individualized interventions for post-procedural recurrence.

## 2 Methods

### 2.1 Study population

The study adhered to the Declaration of Helsinki and received ethics approval from the Hospital’s Research Ethics Committee (Ref: KY2022K028). We prospectively enrolled patients who accepted treatment for AF at the Second Hospital of Tianjin Medical University between March 2022 and November 2022, utilising CBA. All enrolled patients have signed an informed consent form. Exclusion criteria included patients who did not undergo CBA, those with severe heart failure, serious infectious diseases, malignant tumours, and autoimmune diseases. Clinical, laboratory, and echocardiographic baseline data were collected from the medical record system. Following an overnight fast, venous blood samples were drawn before surgery and analysed within 1–2 hours using flow cytometry to assess the content of various T cell subsets and the expression of IR on exhausted T cells.

### 2.2 Cryoballoon ablation procedure

Seasoned electrophysiologists performed CBA. The type of anaesthesia-either local or general-was determined by the operator based on the patient’s overall condition. During the procedure, the patient’s electrocardiogram and vital sign parameters were continuously monitored. Specific surgical details were consistent with those described in previous research studies (28).

### 2.3 Flow cytometry detection

Human T cell subsets and IRs were detected and assessed using a modified version of previously published protocols (29). Briefly, whole blood samples were collected at admission from the antecubital vein of each subject using an EDTA anticoagulant-containing tube. 30 µl whole blood was incubated with FC receptor blocking reagent (Biolegend) to avoid Fc receptor-mediated nonspecific staining, then an antibody mixture containing 25µl staining buffer and antibody mix (APC-CY7 anti-human CD4, AF700 anti-human CD8a, BV421 anti-human CD197, PE-CY7 anti-human CD28, FITC anti-human CD57, PE anti-human CD95, BV 510 anti-human CD45RA, BV 650 anti-human CD27, BV 510 anti-human PD-1, PerCP-CY5.5 anti-human KLRG1, PE-CY5 anti-human Tim-3, and PE/Dazzle™ 594 anti-human LAG-3) for 15 minutes at room temperature and kept protected from light. Antibodies were purchased from Biolegend. Then, the volume was brought up to 400 µl with red blood cell lysis buffer and incubated for 10 minutes. Cells were analysed using a BD FACSCelesta flow cytometer, and data were analysed with FlowJo software. We determined the absolute cell count using Precision Count Beads (BioLegend, Cat# [424902]) according to the manufacturer’s instructions. The gating strategy for identifying T cell subsets and geometric mean of fluorescence intensity (MFI) of IRs is presented in **Supplementary Table 1**, **Supplementary Figures 1**, **S2**.

### 2.4 Follow-up

After CBA, follow-up appointments were scheduled at 3-month intervals or earlier if symptoms occurred. The primary clinical endpoint was AF recurrence, defined as sustained atrial arrhythmia lasting more than 30 seconds, as confirmed by surface electrocardiogram or long-term Holter monitoring.

### 2.5 Statistical analysis

The Shapiro-Wilk test was used to assess the normality of the data distribution. Continuous variables were represented as mean ± standard deviation or median (P25; P75), while categorical variables were presented as number (%). Differences in continuous variables were compared using Student’s t-test or the Mann-Whitney U test, whereas categorical variables were compared using the chi-square test or Fisher’s exact test.

LASSO regression analysis was performed on clinical baseline data, echocardiographic data, and laboratory data. Similarly, LASSO regression was applied to select flow cytometry variables.

Subsequently, the candidate risk factors identified by LASSO regression are integrated into a multivariate Cox model to further validate independent risk factors. The hazard ratio (HR) and 95% confidence interval (95% CI) for each variable are calculated, with statistical significance defined as P<0.05. To evaluate the impact of different risk factors on the time to event occurrence, we performed Kaplan-Meier analysis on key risk factors (MV Vmax, MCV, γ-GGT, glucose, PD-1 of CD8^+^ Tnaive, KLRG1 of CD8^+^ Tcm). Kaplan-Meier survival curves were plotted to visually present the differences in recurrence risk among different subgroups, and statistical significance between subgroups was determined by the Log-rank test.

Two new predictive models for the recurrence of AF after CBA were developed based on the results identified through multivariate analysis. Model 1, which only includes traditional factors (MCV, γ-GGT, glucose, and MV Vmax); and Model 2, which includes traditional risk factors along with data related to exhausted T cells (KLRG1 of CD8^+^ Tcm, and PD-1 of CD8^+^ Tnaive). Both models were evaluated using time-dependent receiver operating characteristic (ROC) curves, concordance index (C-index), calibration curves, and clinical applicability analyses in R. The results of the Cox regression model were visualised using a nomogram, presenting the weight of each independent risk factor intuitively through a scoring system. All statistical tests were two-sided, with P<0.05 considered statistically significant. All statistical analyses and graphing were conducted using the R software.

## 3 Results

### 3.1 Study population characteristics

**Figure 1:**
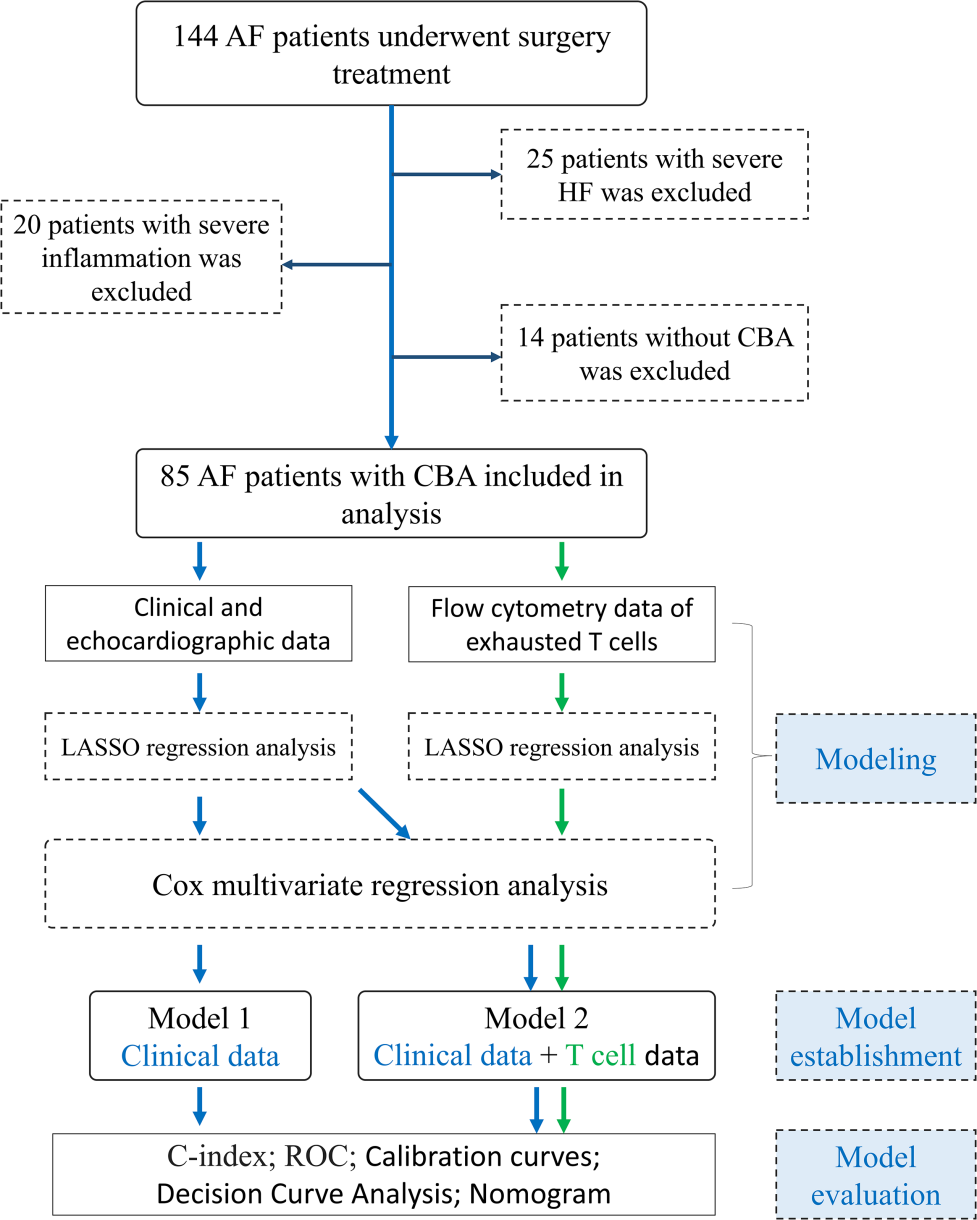
Flow diagram of study design.

The study included 85 patients undergoing CBA (51.8% male, 48.2% female, average age 66.5 ± 9.47 years) (**Figure 1**). Over a mean follow-up duration of 397 ± 311 days, 35 patients (41%) experienced a recurrence of AF. The patients were divided into two groups: recurrence and non-recurrence. In the recurrence group, glucose levels and mitral valve maximum blood flow velocity were significantly higher (p < 0.05), whereas in the non-recurrence group, activated partial thromboplastin time (APTT), mean corpuscular volume (MCV), mean corpuscular hemoglobin concentration (MCHC), and indirect bilirubin content were elevated (p < 0.05). No significant differences were observed in other baseline parameters between the two groups (**Table 1**).

**Table 1:** Baseline characteristics of the study population.

Variable	ALL patients *N=85*	AF unrecurrence group *N=50*	AF recurrence group *N=35*	*p*
CHA_2_DS_2_VASc score	2.00 [1.00;4.00]	2.00 [1.00;4.00]	3.00 [1.50;4.00]	0.344
CHADS_2_ score	1.00 [0.00;2.00]	1.00 [0.00;2.00]	1.00 [1.00;2.00]	0.131
sex				0.866
Female	41 (48.2%)	25 (50.0%)	16 (45.7%)	
Male	44 (51.8%)	25 (50.0%)	19 (54.3%)	
Age (year)	66.5 (9.47)	65.2 (9.38)	68.4 (9.41)	0.128
smoke				1.000
No	55 (64.7%)	32 (64.0%)	23 (65.7%)	
Yes	30 (35.3%)	18 (36.0%)	12 (34.3%)	
drink				1.000
No	60 (70.6%)	35 (70.0%)	25 (71.4%)	
Yes	25 (29.4%)	15 (30.0%)	10 (28.6%)	
AF type				0.607
Paroxysmal AF	69 (81.2%)	42 (84.0%)	27 (77.1%)	
Persistent AF	16 (18.8%)	8 (16.0%)	8 (22.9%)	
Heart Failure				1.000
No	80 (94.1%)	47 (94.0%)	33 (94.3%)	
Yes	5 (5.88%)	3 (6.00%)	2 (5.71%)	
PCI				0.695
No	78 (91.8%)	45 (90.0%)	33 (94.3%)	
Yes	7 (8.24%)	5 (10.0%)	2 (5.71%)	
Valvular disease				0.695
No	78 (91.8%)	45 (90.0%)	33 (94.3%)	
Yes	7 (8.24%)	5 (10.0%)	2 (5.71%)	
Grade of hypertension				0.277
Without hypertension	34 (40.0%)	24 (48.0%)	10 (28.6%)	
Grade 1	2 (2.35%)	1 (2.00%)	1 (2.86%)	
Grade 2	21 (24.7%)	10 (20.0%)	11 (31.4%)	
Grade 3	28 (32.9%)	15 (30.0%)	13 (37.1%)	
Diabetes				0.852
No	70 (82.4%)	42 (84.0%)	28 (80.0%)	
Yes	15 (17.6%)	8 (16.0%)	7 (20.0%)	
Hyperuricemia				1.000
No	80 (94.1%)	47 (94.0%)	33 (94.3%)	
Yes	5 (5.88%)	3 (6.00%)	2 (5.71%)	
Hyperlipidemia				0.731
No	76 (89.4%)	44 (88.0%)	32 (91.4%)	
Yes	9 (10.6%)	6 (12.0%)	3 (8.57%)	
Old cerebral infarction				0.734
No	75 (88.2%)	45 (90.0%)	30 (85.7%)	
Yes	10 (11.8%)	5 (10.0%)	5 (14.3%)	
Renal insufficiency				0.399
No	80 (94.1%)	48 (96.0%)	32 (91.4%)	
Yes	5 (5.88%)	2 (4.00%)	3 (8.57%)	
prothrombin time(s)	13.1±3.33	13.3 ±3.55	12.7 ±3.00	0.379
Prothrombin time activity (%)	92.9 ±24.3	91.7 ±23.3	94.6 ±25.9	0.597
INR	1.11 ±0.32	1.13 ±0.36	1.09 ±0.27	0.589
activated partial thromboplastin time(s)	32.5 ±8.50	34.4 ±8.59	29.9 ±7.78	0.016
Fibrinogen(g/L)	3.11 [2.64;3.41]	3.00 [2.60;3.37]	3.17 [2.85;3.52]	0.097
ProthrombinTime(s)	19.4 ±14.2	20.4 ±18.6	17.9 ±1.22	0.363
D-Dimer(mg/L)	429 [261;533]	418 [263;533]	447 [274;533]	0.733
NT-ProBNP(ng/L)	746 ±1083	686 ±947	832 ±1262	0.564
White blood cell count(10^9^/L)	6.55 ±1.65	6.64 ±1.88	6.44 ±1.25	0.555
Neutrophil percentage(%)	64.2 ±8.11	64.5 ±8.31	63.6 ±7.91	0.619
Lymphocyte percentage(%)	26.3 [22.0;30.8]	26.3 [21.2;30.1]	26.3 [23.2;33.2]	0.485
Monocytes percentage(%)	6.60 [5.30;7.10]	6.60 [5.23;7.27]	6.60 [5.40;6.75]	0.561
Eosinophil percentage(%)	2.18 ±1.87	2.12 ±1.75	2.26 ±2.05	0.739
Basophils percentage(%)	0.45 ±0.20	0.46 ±0.23	0.43 ±0.14	0.394
Neutrophil count(10^9^/L)	4.30 ±1.39	4.40 ±1.57	4.15 ±1.10	0.401
Lymphocyte count(10^9^/L)	1.69 [1.37;1.98]	1.67 [1.33;1.97]	1.69 [1.48;1.93]	0.707
Monocytes count(10^9^/L)	0.43 [0.32;0.49]	0.43 [0.32;0.52]	0.43 [0.32;0.47]	0.364
Eosinophil count(10^9^/L)	0.14 ±0.11	0.13 ±0.10	0.14 ±0.12	0.768
Basophils count(10^9^/L)	0.03 ±0.01	0.03 ±0.01	0.03 ±0.01	0.400
Red blood cell count(10^9^/L)	4.41 ±0.59	4.41 ±0.59	4.42 ±0.60	0.891
Haemoglobin (g/L)	135 ±18.2	136 ±17.0	133 ±19.8	0.358
Hematocrit(L/L)	40.4 ±5.24	40.8 ±5.13	39.7 ±5.40	0.348
Mean corpuscular volume(fL)	91.7 ±5.10	92.9 ±4.97	90.0 ±4.83	0.007
Mean corpuscular hemoglobin content(pg)	30.6 ±2.01	31.1 ±1.77	30.0 ±2.20	0.024
Width of erythrocyte volume distribution-CV(%)	13.3 ±0.96	13.1 ±0.56	13.5 ±1.33	0.152
Mean corpuscular hemoglobin concentration(g/L)	334 ±7.58	334 ±6.49	333 ±8.98	0.583
Platelet count(10^9^/L)	210 [183;220]	202 [182;222]	213 [186;214]	0.986
Platelet crit	0.21 ±0.05	0.22 ±0.06	0.21 ±0.04	0.246
Mean platelet volume(fL)	10.1 ±1.00	10.1 ±0.88	10.0 ±1.17	0.896
Width of erythrocyte volume distribution-SD(fL)	43.7 ±3.07	44.0 ±3.14	43.2 ±2.94	0.220
Percentage of large platelets(%)	26.3 ±6.74	26.2 ±6.14	26.4 ±7.60	0.885
Platelet distribution width(fL)	13.6 ±2.59	13.3 ±2.53	14.1 ±2.63	0.159
Large platelet count(10^9^/L)	55.3 [46.0;61.0]	53.0 [46.0;61.0]	55.3 [46.0;61.5]	0.535
Na^+^(mmol/l)	141 [140;143]	141 [140;143]	140 [140;142]	0.319
K^+^(mmol/l)	4.10 [3.80;4.50]	4.10 [3.80;4.40]	4.20 [4.00;4.60]	0.114
Cl^-^(mmol/l)	107 ±2.81	108 ±2.80	107 ±2.79	0.164
Carban dioxide-combining power(mmol/l)	24.4 ±1.73	24.3 ±1.58	24.5 ±1.94	0.720
Total protein(g/L)	69.5 ±9.80	69.9 ±12.1	68.9 ±4.97	0.613
Albumin (g/L)	41.8 ±3.50	41.6 ±3.85	42.0 ±2.96	0.579
Globulin (g/L)	27.4 [24.8;28.2]	26.9 [24.1;28.1]	27.6 [26.2;27.9]	0.443
Albumin / Globulin	1.58 ±0.24	1.57 ±0.28	1.60 ±0.16	0.471
Alanine aminotransferase(U/L)	20.2 ±10.4	18.7 ±9.17	22.4 ±11.8	0.129
Aspartate aminotransferase(U/L)	18.5 ±5.25	18.1 ±3.97	19.0 ±6.69	0.452
Total bilirubin(umol/l)	15.5 ±5.64	16.2 ±6.22	14.5 ±4.59	0.153
AST / ALT	1.14 [0.91;1.22]	1.16 [0.97;1.25]	1.08 [0.76;1.22]	0.163
Direct bilirubin(umol/l)	4.61 ±2.23	4.56 ±2.40	4.67 ±1.99	0.821
Indirect bilirubin(umol/l)	10.9 ±4.23	11.7 ±4.89	9.84 ±2.79	0.034
Alkaline phosphatase (U/L)	72.0 ±16.1	70.4 ±15.9	74.3 ±16.2	0.280
γ- glutamyltransferase(U/L)	29.9 [20.1;31.2]	26.4 [18.4;31.2]	31.2 [23.0;32.0]	0.053
Urea (mmol/l)	6.45 ±2.11	6.23 ±2.07	6.78 ±2.14	0.242
Creatinine (umol/l)	86.2 ±58.7	76.2 ±19.7	101 ±87.1	0.113
Glucose (mmol/l)	6.39 ±1.61	6.04 ±1.18	6.90 ±1.98	0.026
Uric acid (umol/l)	347 ±79.1	343 ±82.3	351 ±75.3	0.652
Creatine kinase(U/L)	81.6 ±29.8	80.5 ±28.0	83.1 ±32.6	0.703
Creatinekinase-MB(U/L)	11.4 ±6.90	12.1 ±6.70	10.3 ±7.14	0.246
Total cholesterol(mmol/l)	4.42 [3.67;5.08]	4.41 [3.43;5.04]	4.42 [4.37;5.08]	0.182
Triglycerides(mmol/l)	1.41 ±0.60	1.40 ±0.63	1.43 ±0.57	0.811
High density lipoprotein cholesterol(mmol/l)	1.19 ±0.23	1.17 ±0.25	1.23 ±0.21	0.283
Low-density lipoprotein-cholesterol(mmol/l)	2.79 ±0.82	2.73 ±0.86	2.88 ±0.75	0.394
Very low density lipoprotein cholesterol(mmol/l)	0.45 ±0.18	0.45 ±0.20	0.46 ±0.17	0.801
Triiodothyronine (pmol/l)	4.36 [4.10;4.76]	4.36 [4.12;4.75]	4.36 [3.99;4.72]	0.805
Free thyroxine(pmol/l)	17.4 ±2.67	17.1 ±3.06	17.8 ±1.99	0.235
thyroid stimulating hormone(uIU/ml)	2.82 ±1.56	2.82 ±1.63	2.82 ±1.50	0.986
Aortic Diameter(mm)	17.2 ±2.61	17.3 ±2.51	17.1 ±2.78	0.843
Mitral Velocity time integral(cm)	21.6 ±6.70	20.7 ±6.08	22.9 ±7.38	0.146
Mean mitral valve flow velocity(cm/s)	49.0 [39.0;59.0]	47.5 [38.0;57.8]	49.9 [43.0;61.0]	0.11
Mitral valve maximum blood flow velocity(cm/s)	91.5 ±25.5	86.3 ±19.5	98.8 ±31.0	0.039
Pulmonary artery diameter(mm)	22.6 ±2.65	22.3 ±2.57	22.9 ±2.76	0.336
Left ventricular posterior wall thickness(mm)	9.19 ±1.62	9.23 ±1.60	9.13 ±1.66	0.779
Right ventricular anterior wall thickness(mm)	3.49 ±0.40	3.53 ±0.37	3.44 ±0.43	0.301
Interventricular Septal Thickness at Diastole(mm)	9.50 ±2.27	9.60 ±2.51	9.35 ±1.89	0.606
Right ventricularend-diastolic dimension(mm)	21.4 ±2.37	21.5 ±2.72	21.3 ±1.81	0.605
Left ventricularend-systolic dimension(mm)	26.4 [23.8;29.7]	25.6 [23.7;29.0]	27.4 [24.3;30.6]	0.177
Ejection fraction(%)	61.3 ±6.46	62.1 ±6.24	60.0 ±6.64	0.144
Left ventricularend-diastolic dimension(mm)	47.9 ±4.45	47.7 ±4.53	48.1 ±4.40	0.665
Aorta Velocity time integral(cm)	25.2 ±6.98	25.6 ±6.43	24.8 ±7.76	0.615
Mean aortic blood flow velocity(cm/s)	86.4 ±21.1	87.8 ±18.6	84.4 ±24.4	0.491
Aortic maximum blood flow velocity(cm/s)	122 ±28.6	123 ±27.6	120 ±30.3	0.692
Left atrium diameter(mm)	42.3 ±5.33	41.9 ±5.46	42.8 ±5.17	0.455
Tricuspid regurgitation velocity(cm/s)	273 [265;273]	273 [259;273]	273 [270;273]	0.522
Tricuspid regurgitation pressure (mmHg)	29.8 [27.0;29.8]	29.8 [26.2;29.8]	29.8 [28.9;29.9]	0.255
Right atrium long diameter(mm)	48.3 ±5.65	48.3 ±5.52	48.4 ±5.91	0.932
Right atrial transverse diameter(mm)	37.0 ±4.95	36.8 ±4.82	37.3 ±5.19	0.644
Pulmonary artery systolic pressure(mmHg)	33.3 [32.0;33.3]	33.3 [29.2;33.3]	33.3 [32.5;33.3]	0.452
Inferiorvenacava (mm)	14.9 ±3.42	15.2 ±3.37	14.6 ±3.52	0.440
Aortic Annuals Diameter(mm)	24.8 ±2.32	24.6 ±1.93	25.1 ±2.78	0.365

### 3.2 Flow cytometry detection result

In the recurrence group, the levels of KLRG1 on CD4^+^ Tem, CD4^+^ Temra and CD8^+^ Tnaive, the levels of CD57 of CD8^+^ Tem were elevated. CD4^+^ T naive count and percentage were elevated (p < 0.05). No significant differences were observed in the remaining parameters between the two groups (**Supplementary Table 2**). The histograms of mean fluorescence intensity for IRs of T cell subsets are now presented in **Supplementary Figure 2**.

### 3.3 Model building

#### 3.3.1 Risk factors screening

LASSO regression analysis was performed on clinical baseline data, echocardiographic data, and laboratory data, and the results showed that mitral valve maximum velocity (MV Vmax), mean corpuscular volume (MCV), γ-Glutamyl Transferase (γ-GGT), creatinine and glucose were associated with recurrent AF after CBA. Meanwhile, LASSO regression analysis of data related to exhausted T cells KLRG1 of CD8^+^ Tcm, PD-1 of CD8^+^ Tnaive, and KLRG1 of CD4^+^ Temra demonstrated that were associated with recurrent AF after CBA (**Supplementary Figures 3**, **4**).

**Figure 2:**
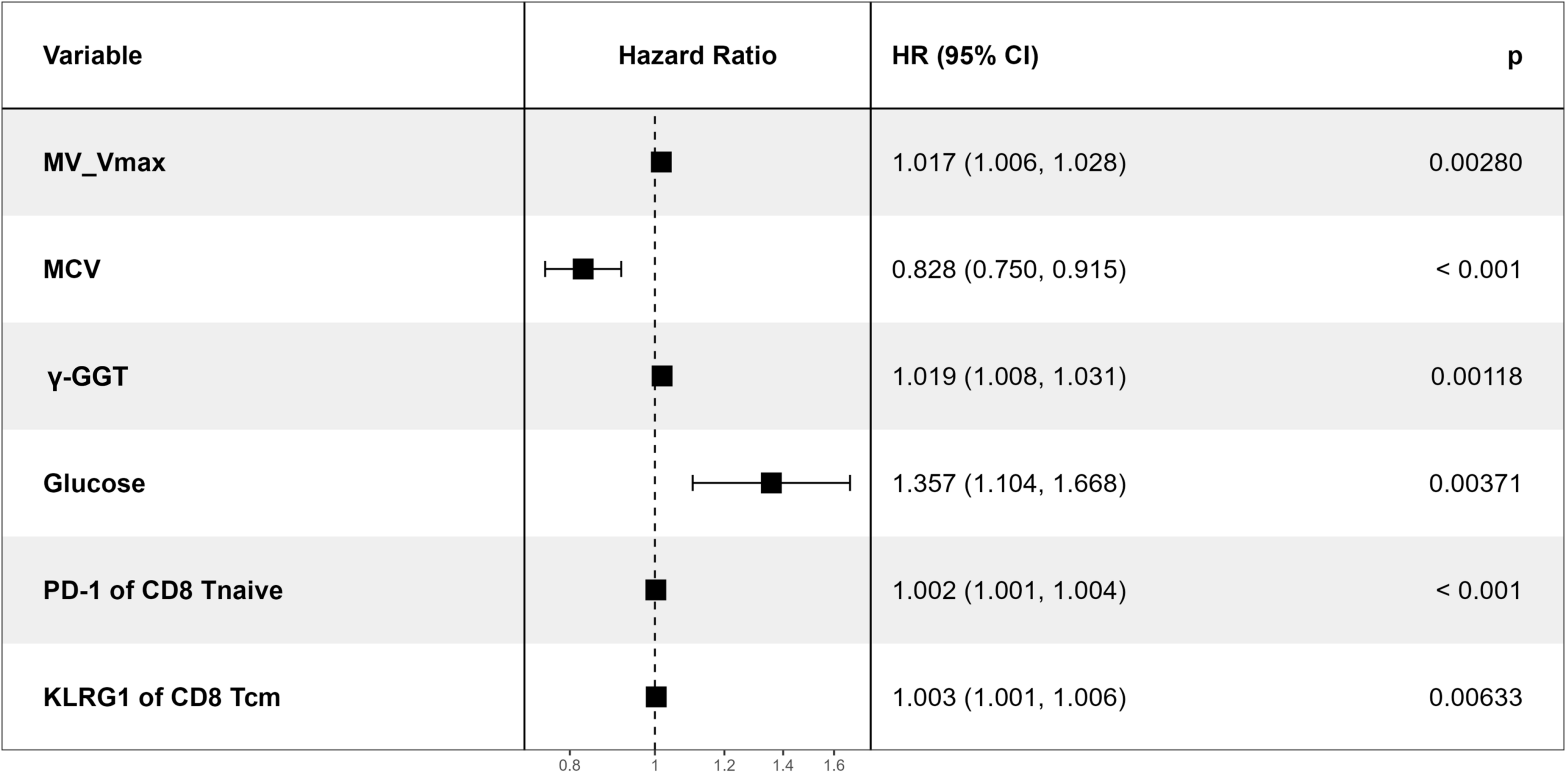
Forest plot of the results of the multivariate analysis. X-axis: hazard ratio (HR), reflecting the strength of association between each variable and AF recurrence. Y-axis: variables included in multivariable analysis. Error bars: 95% confidence interval (95% CI). An association was statistically significant if error bars did not cross HR = 1.

Candidate variables screened by LASSO regression were integrated and incorporated into a multivariate Cox proportional hazards regression model to verify and identify independent risk factors for AF recurrence. Cox multivariate regression analysis revealed that MV Vmax (HR: 1.017, 95%CI: 1.006-1.028, P = 0.0028), MCV (HR: 0.828, 95%CI: 0.750-0.915, P<0.001), γ-GGT (HR: 1.019, 95%CI:1.008-1.031, P = 0.0012), glucose (HR: 1.357, 95%CI: 1.104-1.668, P = 0.0037), PD-1 of CD8^+^ Tnaive (HR: 1.002, 95%CI: 1.001-1.004, P<0.001), and KLRG1 of CD8^+^ Tcm (HR:1.003, 95%CI: 1.001-1.006, P = 0.0063) were independent risk factors for AF recurrence after CBA. (**Figure 2**). The histograms of mean fluorescence intensity for T cell subsets and inhibitory molecules included in the predictive model are now presented in **Supplementary Figure 5**.

**Figure 3:**
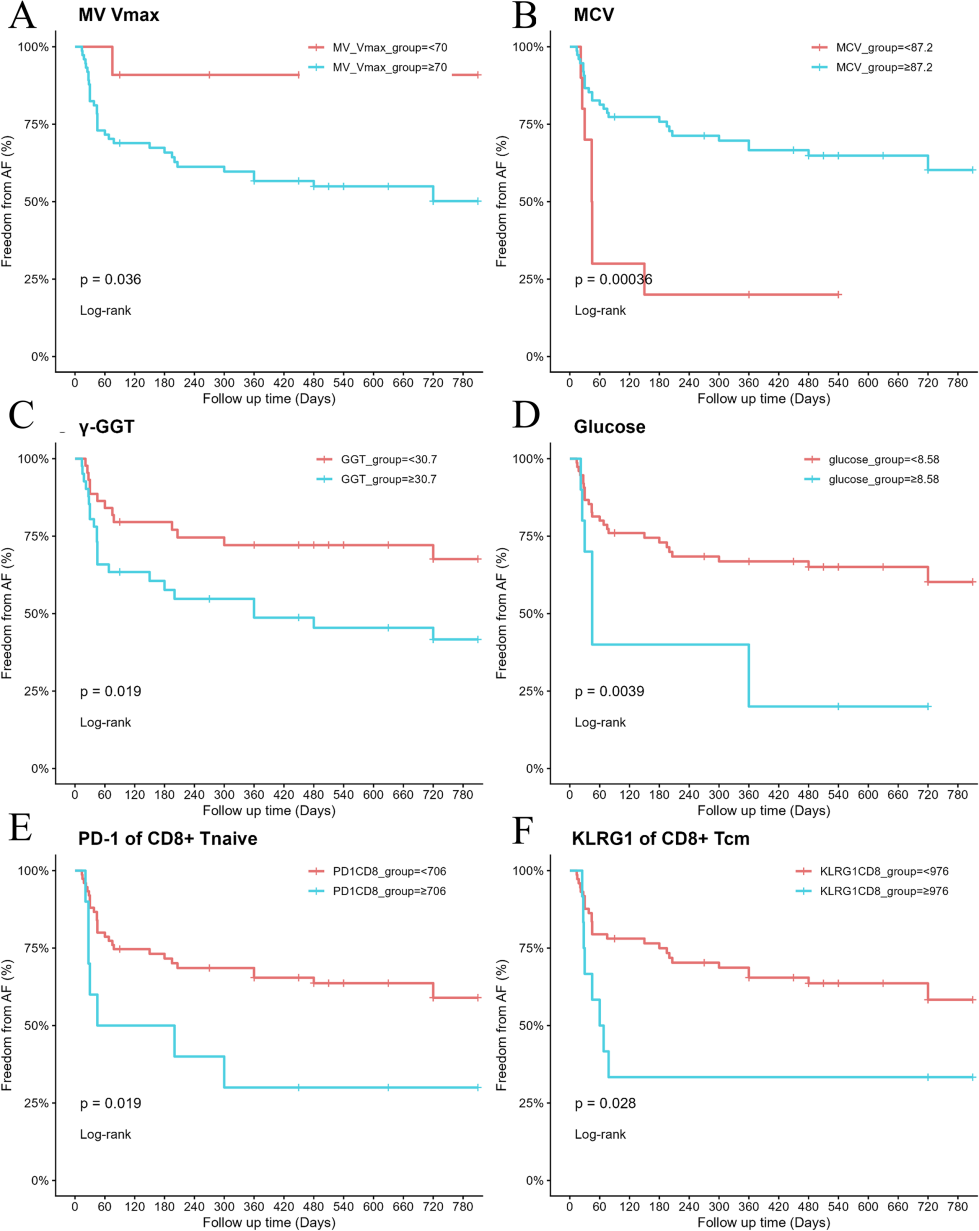
Kaplan-Meier survival analysis of key risk factors for AF recurrence after CBA: MV Vmax **(A)**, MCV **(B)**, γ-GGT **(C)**, glucose **(D)**, PD-1 of CD8^+^ Tnaive **(E)**, KLRG1 of CD8^+^ Tcm **(F)**. Y-axis: AF recurrence-free survival rate (0-1.00); X-axis: follow-up time (unit: days).

In order to assess the effect of different risk factors on the time to event occurrence, we conducted Kaplan-Meier analysis on key risk factors. The results indicated that patients with the following characteristics had a significantly higher risk of recurrent AF post-CBA during follow-up: MV Vmax ≥ 70 (**Figure 3A**), MCV < 87.2 fL (**Figure 3B**), γ-GGT ≥ 30.7 U/L (**Figure 3C**), glucose ≥ 8.58 mmol/L (**Figure 3D**), PD-1 MFI of CD8^+^ Tnaive ≥ 706 (**Figure 3E**), KLRG1 MFI of CD8^+^ Tcm > 976 (**Figure 3F**).

#### 3.3.2 Model development

Based on the results of multivariate analysis, six variables (MV Vmax, MCV, γ-GGT, glucose, PD-1 MFI of CD8^+^ Tnaive and KLRG1 MFI of CD8^+^ Tcm) were included in the predictive model (**Figure 2**). To investigate the role of T cell IRs in AF recurrence, we established two models: Model 1, comprising traditional clinical data (MV Vmax, MCV, γ-GGT, glucose), and Model 2, which added T cell IRs to the traditional clinical data (MV Vmax, MCV, γ-GGT, glucose, PD-1 MFI of CD8^+^ Tnaive and KLRG1 MFI of CD8^+^ Tcm).

#### 3.3.3 Model evaluation

**Figure 4:**
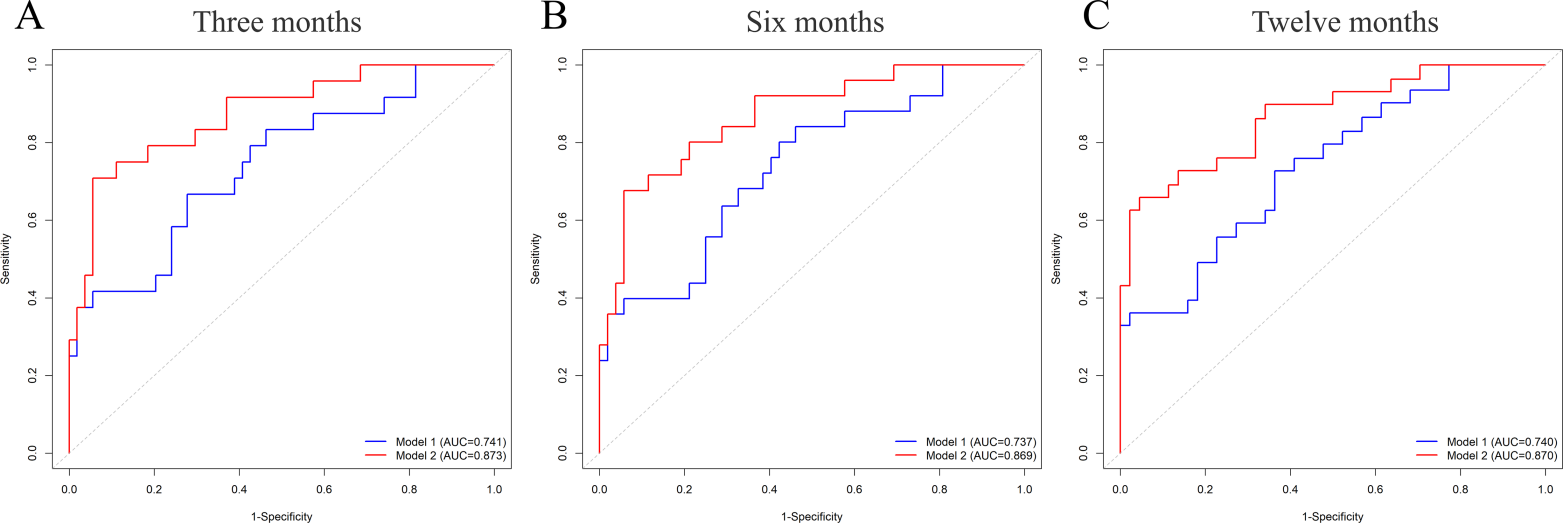
Time-dependent ROC curves of Model 1 and 2 for predicting AF recurrence after CBA. Y-axis: true positive rate (sensitivity); X-axis: false positive rate (1-specificity). Higher AUC (closer to 1) indicates better predictability. The time-dependent ROC curves of these two models at 3 months **(A)**, 6 months **(B)**, and 12 months **(C)** demonstrated that the clinical application value of Model 2 was superior to that of Model 1. Model 1: MCV, γ-GGT, glucose, and MV Vmax. Model 2: MCV, γ-GGT, glucose, MV Vmax, KLRG1 of CD8^+^ Tcm, and PD-1 of CD8^+^ Tnaive.

**Figure 5:**
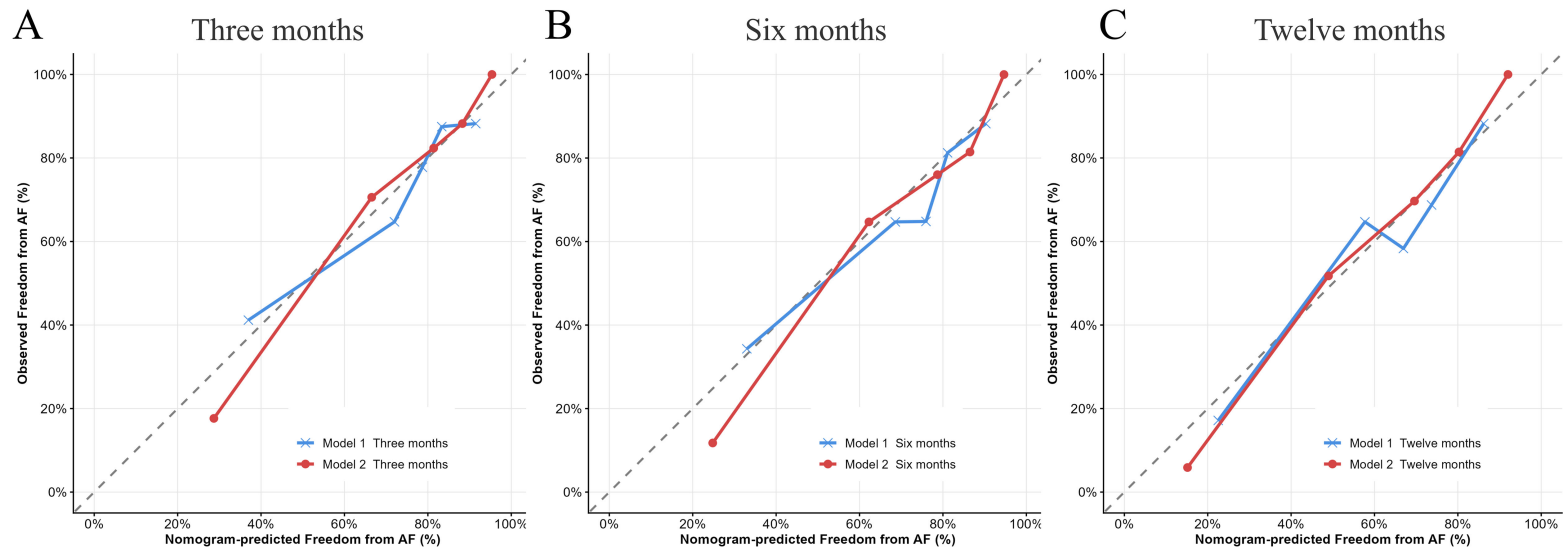
Calibration curves of Model 1 and Model 2 for AF recurrence after CBA. X-axis: predicted AF recurrence probability; Y-axis: observed AF recurrence probability. The diagonal line (Y=X) represents perfect agreement between predicted and observed probabilities. Calibration curves at 3 months **(A)**, 6 months **(B)**, and 12 months **(C)** showed that Model 2 exhibited better calibration than Model 1. Model 1: MCV, γ-GGT, glucose, and MV Vmax. Model 2: MCV, γ-GGT, glucose, MV Vmax, KLRG1 of CD8^+^ Tcm, and PD-1 of CD8^+^ Tnaive.

**Figure 6:**
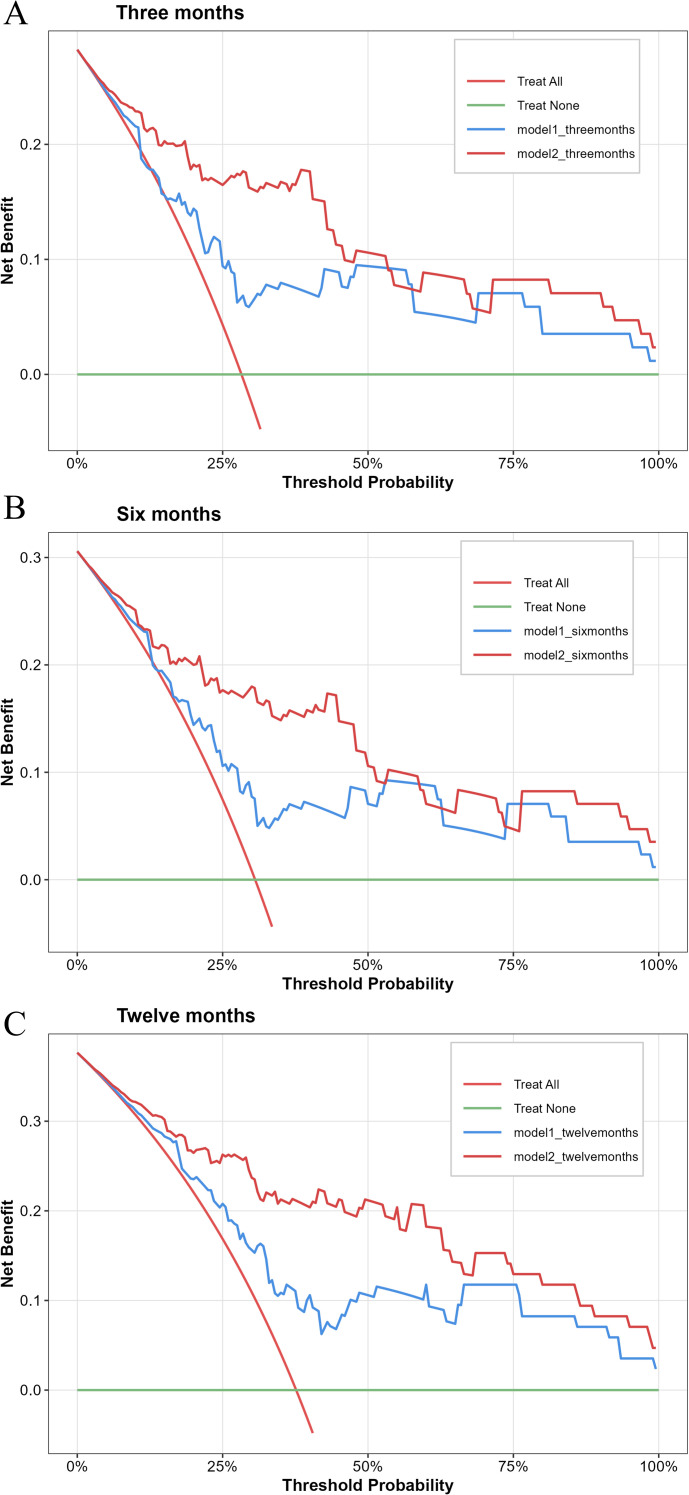
Decision curve analysis (DCA) of Model 1 and Model 2 for predicting AF recurrence after CBA. X-axis: clinical decision threshold probability; Y-axis: net benefit of risk prediction (higher values indicate better clinical utility). Solid red line: treating all patients (net benefit decreases with increasing threshold); solid green line: treating no patients (net benefit = 0). DCA at 3 months **(A)**, 6 months **(B)**, and 12 months **(C)** showed that Model 2 had superior clinical utility compared with Model 1. Model 1: MCV, γ-GGT, glucose, and MV Vmax. Model 2: MCV, γ-GGT, glucose, MV Vmax, KLRG1 of CD8^+^ Tcm, and PD-1 of CD8^+^ Tnaive.

To evaluate the predictive efficacy of the two models for AF recurrence, their discrimination, calibration, and clinical applicability were compared. The C-index for Models 1 and 2 was 0.712 (95% CI: 0.612-0.773) and 0.82 (95% CI: 0.723-0.864), respectively. The AUC for predicting AF recurrence within 3 months post-CBA were 0.741 and 0.873 for Models 1 and 2, respectively (**Figure 4A**). For the 6-month post-CBA prediction, the AUCs were 0.737 and 0.869 (**Figure 4B**), and for the 12-month post-CBA prediction, the AUCs were 0.740 and 0.870, respectively (**Figure 4C**). These results indicate that Model 2 showed significantly higher predictive capabilities than Model 1. The calibration curves for both models demonstrated that Model 2 had superior calibration, with its predicted probabilities more closely aligned with the actual observed probabilities (**Figure 5**). Decision Curve Analysis (DCA) further assessed the models’ clinical utility, revealing that Model 2 provided greater clinical value than Model 1 (**Figure 6**).

In summary, incorporating risk factors related to exhausted T cells into traditional risk factors (Model 2) significantly enhanced the model’s ability and accuracy in predicting AF recurrence.

#### 3.3.4 Construction of the optimal model nomogram

**Figure 7:**
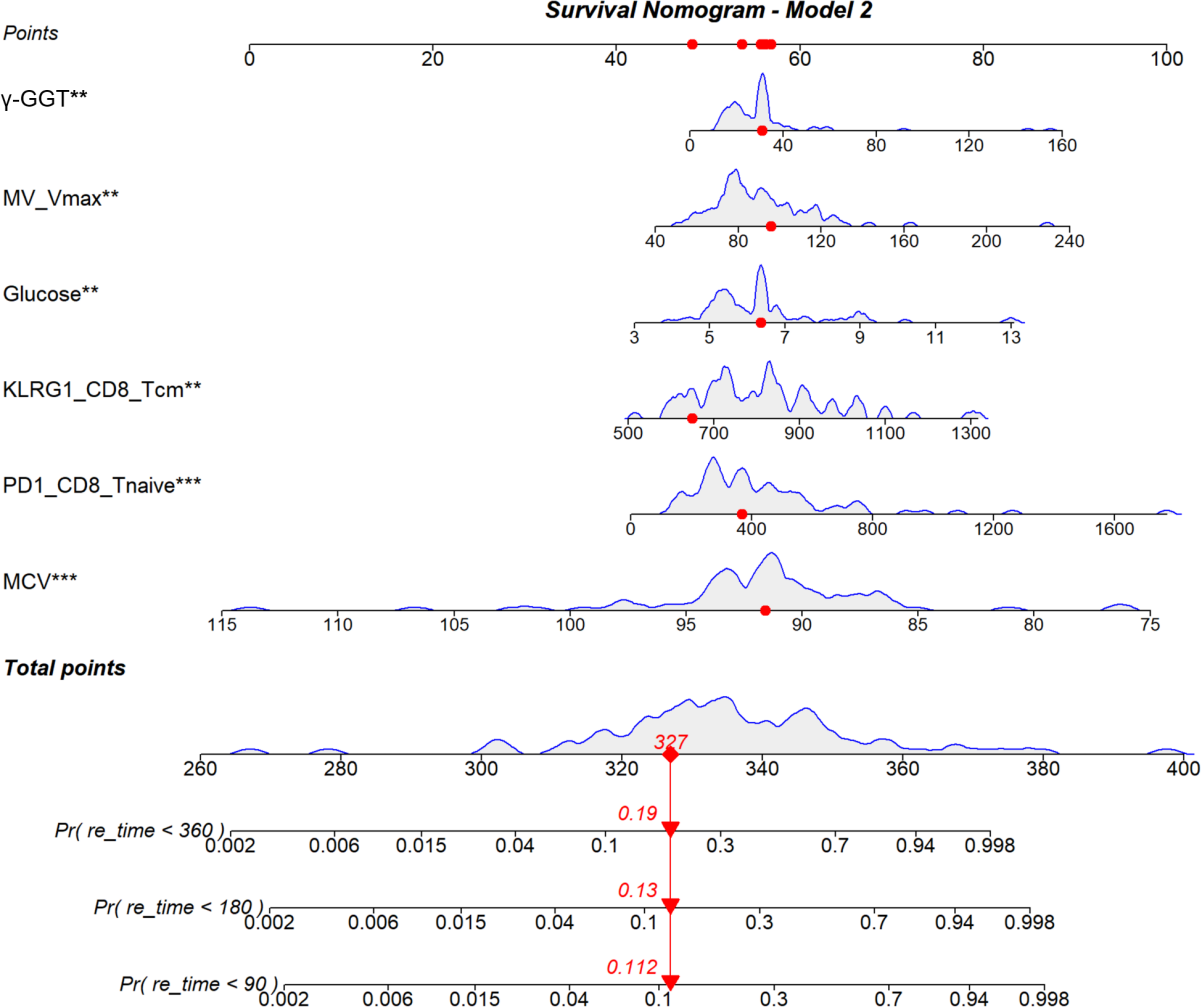
Nomogram for predicting AF recurrence after CBA based on the Cox proportional hazards model. Included variables: MCV, γ-GGT, glucose, MV Vmax, KLRG1 of CD8^+^ Tcm, and PD-1 of CD8^+^ Tnaive. Points for each variable are shown on the axis; the total score is the sum of all points. A vertical line from the total score predicts individual AF recurrence risk following CBA.

Based on the multivariable Cox regression results, we constructed a novel nomogram to predict AF recurrence after CBA (**Figure 7**).

## 4 Conclusion

This study indicates that, in addition to traditional risk factors such as γ-GGT, glucose, MCV, MV Vmax, and the expression level of KLRG1 of CD8^+^ Tcm and PD-1 of CD8^+^ Tnaive are novel independent risk factors for AF recurrence after CBA and play a significant role in early recurrence of AF; we have constructed a new predictive nomogram for AF recurrence using the Cox proportional risk model and the inclusion of KLRG1^+^ and PD-1^+^ T cell-related subgroups significantly enhances the model’s predictive accuracy, calibration, and clinical applicability.

## 5 Discussion

Numerous factors contribute to the recurrence of AF following CBA. Yet, to the best of our knowledge, no studies have examined the impact of exhausted T cells on the recurrence of AF post-CBA. In this study, we observed differences in the levels of various T cell subsets and the expression levels of IR on exhausted T cells between patients with and without AF recurrence after CBA. To investigate the impact of these differences on AF recurrence, we conducted a follow-up study and employed the multivariate Cox proportional hazards regression model to identify risk factors for AF recurrence and establish a novel predictive model.

Our Cox multivariate analysis revealed that KLRG1 of CD8^+^ Tcm, PD-1 of CD8^+^ Tnaive, MCV, γ-GGT, glucose, and MV Vmax levels were independent risk factors for AF recurrence. Based on the identified risk factors, we constructed two predictive models: Model 1, which only includes traditional factors (MCV, γ-GGT, glucose, and MV Vmax); and Model 2, which includes traditional risk factors along with data related to exhausted T cells (KLRG1 of CD8^+^ Tcm, and PD-1 of CD8^+^ Tnaive). Assessment of both models revealed that Model 2 possesses better predictive capabilities and good calibration, while DCA demonstrated the clinical applicability of Model 2. Existing AF recurrence scoring systems (e.g., CHA_2_DS_2_-VASc) primarily focus on conventional risk factors, including age, sex, and comorbidities, with limited predictive dimensions and modest performance. In this study, we constructed a predictive model incorporating exhausted T cell-related markers, which achieved substantially improved predictive performance. Model 2 (with immune markers) yielded higher C-index and AUC values at 3, 6, and 12 months than Model 1 (conventional factors only), and better agreement between predicted and observed probabilities in calibration curves. DCA confirmed the superior net clinical benefit of the novel model. To our knowledge, this is the first model to integrate immunosenescence as a predictive dimension, addressing the critical gap in current scoring systems left by neglected immune mechanisms. It enables more precise identification of patients at high risk of AF recurrence, reduces both over-treatment and under-diagnosis, and provides evidence for individualized interventions, thereby facilitating optimized clinical decision-making.

Based on Model 2, this study constructed a nomogram integrating traditional clinical factors and exhausted T cell-related immune markers, providing clinicians with a convenient visual predictive tool. For AF patients scheduled for CBA, clinicians can obtain baseline clinical and laboratory indicators (MCV, γ-GGT, glucose, and MV Vmax) through routine examinations, detect the expression levels of KLRG1 (CD8^+^ Tcm) and PD-1 (CD8^+^ Tnaive) on specific T cell subsets by flow cytometry before surgery, calculate the total score according to the nomogram assignment, and then directly read the individual AF recurrence risk probabilities at 3, 6, and 12 months after CBA. This is of great significance for early intervention in subclinical AF recurrence and for improving patients’ long-term prognosis. Currently, flow cytometry has achieved clinical standardization, with clear, reproducible detection protocols, convenient sample collection, and compatibility with the preoperative CBA cycle. Medical institutions without relevant platforms can improve accessibility through regional collaboration. Although it increases detection costs, it can accurately identify high-risk patients, avoiding waste of medical resources and reducing recurrence-related medical expenditures. In summary, T cell immunophenotyping is feasible for clinical application and can maximize its clinical and economic benefits.

KLRG1 is a C-type lectin IR primarily recognised as an inhibitor molecule and a marker of exhausted cells (30). It is expressed on the surface of natural killer (NK) cells and T cells, involving MHC I class molecules. Research on KLRG-1 has mainly focused on cancer-related (31) and immune-related diseases (32), with little investigation in the context of AF. KLRG1 is not only a marker of exhausted T cells (33), but unlike complete functional exhaustion in exhausted T cells, KLRG1^+^ T cells have low proliferation capacity, though they do not necessarily have reduced cytotoxicity or cytokine production (34). Studies on ageing cells with high KLRG1 expression support this theory (35). Recent research has also shown that KLRG1, an exhaustion marker on T cells and NK cells, can be highly expressed in the peripheral blood of patients with chronic inflammation, such as tuberculosis (36, 37), hepatitis B (38, 39), and hepatitis C (40, 41). Studies suggest that KLRG1^+^ CD8^+^ T cells tend to accumulate at inflammatory sites (42) and have a significantly higher ability to produce pro-inflammatory factors than KLRG1^-^ T cells (43). In primary biliary cirrhosis(PBC), KLRG1^+^ T cells are rich in inflammatory cytokines and are highly inflamed, with higher expression of inflammatory chemokine receptors (CCR5 and CX3CR1) (44). Most importantly, KLRG1^+^ memory CD8^+^ T cells can significantly impact the progression and prognosis of diseases (45). Other studies suggest that the expression of KLRG1 on memory cells may depend on exposure to inflammatory signals during CD8^+^ T cells differentiation (46, 47). These findings indicate that KLRG1^+^ T cells have pro-inflammatory potential, and the expression level of KLRG1 may reflect the degree of systemic inflammation to some extent.

Advancing age can impair the innate immune response, contributing to chronic low-grade inflammation, a process known as inflammaging (48, 49). Studies have shown that the expression of KLRG1 in CD8^+^ T cells increases with age in adults (50, 51). Additionally, highly differentiated KLRG1^+^ lymphocytes promote inflammation and contribute to inflammaging (52), and recent research has further revealed that KLRG1 can identify a subpopulation of regulatory T (Treg) cells with mitochondrial alterations that accumulate with aging in both humans and mice (53). These KLRG1^+^ Treg cells display distinct senescence features including mitochondrial dysfunction, upregulated expression of cell-cycle regulators, genomic DNA damage, and a pro-inflammatory phenotype with reduced *in vivo* suppressive activity (53), which further confirms that KLRG1+ immune cells are key mediators of age-related chronic inflammation and inflammaging. These findings indicate that the expression of KLRG1 in CD8^+^ T cells reflects immune-related ageing, providing a more accurate assessment of the relationship between inflammation, age and disease. Moreover, both inflammation levels and age are independent risk factors for the recurrence of AF after ablation (52). Our results demonstrate that KLRG1 of CD8^+^ Tcm is an independent risk factor for recurrence of AF after CBA. The expression levels of KLRG1 of CD8^+^ Tcm and age were higher in the recurrence group than in the non-recurrence group. Based on these findings and existing evidence, we hypothesise that the recurrence of AF after CBA may be associated with the pro-inflammatory characteristics of KLRG1^+^T cells, which contribute to chronic low-grade inflammation and inflammaging in the body.

Prior studies have shown that KLRG1^+^ immune cells are associated with fibrosis in various diseases. In PBC patients, a large number of KLRG1^+^ CD8^+^ T cells infiltrate the liver, with increased expression correlating with the severity of liver fibrosis (44). In addition, KLRG1^+^ NK cells have a stronger anti-fibrotic ability compared to KLRG1^-^ NK cells in chronic inflammatory diseases. In systemic sclerosis (SSc) patients, the proportion of KLRG1^+^ immune cells in the skin correlates with the severity of fibrosis, and increased KLRG1 expression alleviates skin fibrosis (54). Based on these findings and our results, we hypothesise that KLRG1^+^ T cells may also contribute to the progression of fibrosis in AF and that their expression levels are positively correlated with myocardial fibrosis in this condition. KLRG1^+^CD8^+^Tcm cells may be involved in the process of myocardial fibrosis through multiple mechanisms. On the one hand, the proinflammatory cytokines secreted by this cell subset can activate cardiac fibroblasts, promote their proliferation and collagen synthesis, and increase the deposition of collagen fibers in myocardial tissues (55). On the other hand, KLRG1^+^CD8^+^Tcm cells can upregulate the expression of fibrosis-related genes (e.g., TGF-β, CTGF) in cardiac fibroblasts through direct cell-cell interactions, thereby further accelerating the progression of myocardial fibrosis (56). Myocardial fibrosis leads to increased stiffness of myocardial tissues, impairs the normal electrical signal conduction between cardiomyocytes, and forms electrical conduction barriers and reentrant circuits, thus elevating the likelihood of AF recurrence (57). However, this hypothesis requires further investigation.

KLRG1^+^CD8^+^Tcm cells may also contribute to the recurrence of AF after CBA by participating in the process of myocardial electrical remodeling, a key pathophysiological mechanism underlying the initiation and maintenance of AF that is mainly characterized by abnormal expression and function of ion channels in cardiomyocytes (58). Proinflammatory cytokines secreted by KLRG1^+^CD8^+^Tcm cells may modulate the expression and activity of ion channels (e.g., sodium, potassium, and calcium channels) in cardiomyocytes, thereby promoting the formation of ectopic pacemakers and the maintenance of reentrant excitation and precipitating the recurrence of AF (59). In addition, following their infiltration into myocardial tissues, KLRG1^+^CD8^+^Tcm cells can directly interact with cardiomyocytes and damage them by releasing cytotoxic substances (e.g., granzymes, perforins), which induce abnormal electrophysiological characteristics in cardiomyocytes, further exacerbating myocardial electrical remodeling, and elevating the risk of AF recurrence (60).

A recent study has revealed a moderate positive correlation between the content of KLRG1^+^ CD8^+^ T cells and disease duration (61). Our data also supports this finding, with a significantly higher proportion of persistent AF in the recurrence group compared to the non-recurrence group (22.9% vs. 16%). This study observed the overall recurrence rate of AF after CBA, without distinguishing between early and late recurrence. As shown by the Kaplan-Meier curve, the steepest slope occurs within 30 days, suggesting that KLRG1 of CD8^+^ Tcm plays a significant role in the early recurrence of AF after ablation. This may be associated with the presence of inflammatory responses and tissue injury in myocardial tissues at the early stage after CBA. KLRG1^+^CD8^+^Tcm cells rapidly infiltrate the myocardium in the early postoperative phase and rapidly induce pathophysiological changes in the heart through the aforementioned inflammation-, fibrosis- and electrical remodeling-related mechanisms, thereby contributing to the early recurrence of AF (62).

In this study, multivariate Cox regression analysis showed for the first time that PD-1 expression on CD8^+^ Tnaive cells acts as an independent risk factor for AF recurrence after CBA. PD-1 is a critical immune checkpoint molecule involved in regulating T cell exhaustion through inhibitory signaling (18). When PD-1 binds its ligands PD-L1 and PD-L2, it recruits phosphatases SHP-1 and SHP-2, which inhibit T cell proliferation, decrease cytokine secretion, and impair effector function (22). Normally, PD-1 expression on CD8^+^ Tnaive cells is low and increases during the early activation phase after antigen exposure (20). In this study, elevated PD-1 levels on CD8^+^ Tnaive cells in the recurrence group suggest ongoing antigenic stimulation or pre-activation, leading to phenotypic changes in naive T cells before full antigen exposure; this mechanism aligns with the chronic inflammatory state characteristic of AF (27).

Tissue injury and repair after CBA release large amounts of damage-associated molecular patterns (DAMPs), initiating acute local and systemic inflammation. This inflammation can increase PD-1 expression on CD8^+^ Tnaive cells (63). Necrosis of cardiomyocytes from cryoablation releases endogenous antigens—such as nucleic acids and proteins—that persistently stimulate CD8^+^ Tnaive cells in peripheral lymphoid organs, leading to premature activation and higher PD-1 expression (23). Chronic low-grade inflammation in AF patients keeps CD8^+^ Tnaive cells in a primed state by activating antigen-presenting cells, including dendritic cells (DCs), which elevates baseline PD-1 expression (24). In our study, the recurrence group had higher blood glucose levels. Hyperglycemia may further raise PD-1 expression on immune cells through oxidative stress and inflammation, acting as a synergistic factor in high PD-1 expression on CD8^+^ Tnaive cells (64).

Persistent activation of PD-1 directly inhibits the proliferation and differentiation potential of CD8^+^ Tnaive cells (65). Tnaive cells, under physiological conditions, differentiate into effector and memory T cells following antigen stimulation, thereby mediating specific immune defense (66). If the PD-1/PD-L1 pathway remains activated, however, it reduces the proliferative capacity of these naive cells and impairs their differentiation into cytotoxic effector CD8^+^ T cells. Consequently, abnormal antigens in myocardial tissue are cleared less efficiently (67). Weakened immune clearance then hampers the removal of aberrant inflammatory and fibrosis-related cells during myocardial injury repair after CBA (68). Ultimately, this accumulation of cells promotes myocardial fibrosis and electrical remodeling (69).

Furthermore, high PD-1 expression in CD8^+^ Tnaive cells induces their premature differentiation into exhausted T cells (70). Previous studies have demonstrated that Tnaive cells bypass the normal effector differentiation stage and directly enter an exhausted state under combined persistent antigen stimulation and PD-1 signaling (7). Although exhausted CD8^+^ T cells still infiltrate myocardial tissue, their ability to secrete pro-inflammatory cytokines such as interferon-γ and tumor necrosis factor-α is markedly reduced, accompanied by attenuated cytotoxicity, rendering them unable to effectively suppress the activation and proliferation of cardiac fibroblasts (71). Meanwhile, exhausted T cells secrete anti-inflammatory cytokines, including transforming growth factor-β, which impairs the function of reparative immune cells such as M2-type macrophages, further disrupting the myocardial immune microenvironment and accelerating myocardial fibrosis (60).

Our results indicate that the MCV is lower in the recurrence group compared to the non-recurrence group. MCV reduction may be due to anaemia, chronic diseases such as inflammation, or oncological diseases, among others (72). However, our cohort excluded patients with severe infectious diseases, autoimmune diseases, or malignant tumours, and baseline data revealed no cases of anaemia. Accordingly, we speculate that this observation is mainly linked to the chronic low-grade inflammatory state in patients with recurrent AF. Inflammation modulates erythropoiesis through several pathways: sustained pro-inflammatory cytokine secretion (e.g., TNF-α, IFN-γ) from KLRG1^+^CD8^+^ Tcm cells in the recurrence group directly suppresses bone marrow erythroid progenitor proliferation and differentiation, delays erythrocyte maturation, and reduces erythropoietin (EPO) synthesis and activity (73). In addition, inflammation elevates hepcidin levels, impairing bone marrow iron transport and utilisation and causing functional iron deficiency (74). This deficiency, without overt anaemia, compromises erythropoiesis and reduces erythrocyte volume. Moreover, chronic cardiovascular comorbidities (e.g., hypertension) may mildly disturb EPO production and erythropoiesis via tissue hypoperfusion and metabolic disorders (75), and these effects can be amplified by inflammation, synergistically lowering MCV. In turn, reduced MCV may weaken myocardial antioxidant capacity, aggravate electrophysiological instability, and increase vulnerability to inflammatory and oxidative stress, thereby promoting AF recurrence (76).

This study confirms that **γ-**GGT is an independent risk factor for AF recurrence after CBA. As a key enzyme in glutathione metabolism, recent studies have established the central role of γ-GGT in the pathophysiology of cardiovascular diseases. γ-GGT catalyzes the hydrolysis of glutathione to cysteinylglycine. This hydrolytic product can induce the oxidation of low-density lipoprotein via an iron-dependent pathway, thereby promoting the generation of reactive oxygen species (ROS) (77). Abnormal accumulation of ROS directly impairs ion channel function in cardiomyocytes, leading to dysregulated expression of proteins closely related to myocardial electrophysiology, including L-type calcium channels and potassium channels. This disrupts the stability of myocardial electrical activity, thereby establishing an important pathological basis for AF recurrence after CBA (78, 79).In addition to oxidative stress, hepatic congestion, a common complication of cardiac dysfunction, may also upregulate γ-GGT expression (80). However, in the present study, the AF recurrence group did not exhibit significant elevations in markers of hepatic dysfunction or cardiac insufficiency, suggesting that isolated hepatic congestion is not the primary cause of increased γ-GGT levels. Therefore, elevated γ-GGT primarily reflects enhanced systemic oxidative stress and inflammation rather than isolated hepatic congestion. By mediating disturbances in glutathione metabolism, γ-GGT participates in myocardial electrical remodeling, thereby increasing the risk of AF recurrence.

Meanwhile, in this study, hyperglycemia in the recurrence group may represent a critical marker of systemic metabolic dysfunction and acute stress. Metabolically, patients with AF often exhibit impaired glucose metabolism. Hyperglycemia induces myocardial metabolic disturbance, elevated oxidative stress, and vascular injury, thereby exacerbating myocardial pathophysiological changes and increasing AF recurrence risk (64). Key mechanisms include insulin resistance and abnormal glycogen metabolism, which impair cardiomyocyte energy supply. Furthermore, hyperglycemia promotes advanced glycation end product (AGE) formation, aggravating myocardial fibrosis and electrical remodeling (81). Additionally, myocardial injury and inflammatory stimulation following CBA may trigger an acute systemic stress response, increasing stress hormone (e.g., cortisol, epinephrine) secretion. These hormones enhance hepatic glycogenolysis and suppress insulin secretion, leading to transient hyperglycemia (63). Thus, hyperglycemia in the recurrence group likely reflects the synergistic effects of postoperative acute stress and underlying metabolic dysfunction, indicating that blood glucose may serve as a simple biomarker for assessing metabolic status and postoperative acute stress in AF patients.

MV Vmax was an independent risk factor for AF recurrence after CBA, primarily by affecting left atrial (LA) hemodynamics and myocardial remodeling. In a prospective study, Klinger et al. (82) demonstrated that a left atrial appendage (LAA) flow velocity ≤ 60 cm/s was associated with a 3-fold increased risk of new-onset AF. Elevated MV Vmax aggravated LA blood stasis and directly reduced LAA flow velocity (82). Such hemodynamic abnormalities not only increased LA pressure load, induced LA enlargement and myocardial hypertrophy, disrupted the uniformity of electrical conduction, and provided an anatomical substrate for re-entry circuit formation [96], but also promoted the development of spontaneous echo contrast (SEC) and raised thrombotic risk. The associated inflammatory response further exacerbated myocardial fibrosis (83). Using 4D flow MRI, Markl et al. (84) confirmed that peri-wall LA stasis was significantly higher in patients with AF than in the central LA region and was positively correlated with LA volume. Increased MV Vmax further aggravated such regional flow abnormalities and contributed to persistent low-velocity zones. This process activated stretch-activated channels in atrial myocytes, leading to increased Ca²^+^ influx and Ca²^+^ overload (85). Ca²^+^ overload not only triggered sarcoplasmic reticulum Ca²^+^ leakage and spontaneous Ca²^+^ release, thereby inducing delayed afterdepolarizations, but also upregulated the expression of fibrosis-related genes, such as transforming growth factor-β, via activation of the calcineurin (CaN) signaling pathway, accelerating myocardial fibrosis (86). Ultimately, a vicious cycle of structural and electrical remodeling promoted AF recurrence (87).

### 5.1 Limitations

This study was a single-centre, small-sample prospective study, and larger, multi-centre data are needed for validation. As a clinical study, it lacks pathological and molecular biology research to explore the deeper intrinsic mechanisms. This study did not conduct in-depth pathological and molecular biological investigations into the potential mechanisms by which exhausted T-cell markers (e.g., KLRG1 of CD8^+^ Tcm, PD-1 of CD8^+^ Tnaive) mediate AF recurrence after CBA. Future *in vitro* and *in vivo* studies are needed to elucidate the specific cellular and molecular pathways underlying T-cell exhaustion in relation to myocardial inflammation, fibrosis, and electrical remodeling during the initiation and progression of AF.

## Data Availability

The raw data supporting the conclusions of this article will be made available by the authors, without undue reservation.

## Supplementary material

Supplementary Figure 1 Example staining and gating. By staining blood cells for CD3, CD8, CD4, CD197, CD95 and CD45RA can differentiate naÏve (TnaÏve, CD45RA^+^ CD197^+^ CD95^−^), effector memory-expressing CD45RA (TEMRA, CD45RA^+^ CD197^−^), central memory (TCM, CD45RA^−^ CD197^+^), memory stem cell (TSCM, CD45RA^+^ CD197^+^ CD95^+^) and effector memory (TEM, CD45RA^−^ CD197^−^) CD4^+^ and CD8^+^ T cells. The mean fluorescence intensity (MFI) of CD27, KLRG1, LAG-3, Tim-3, PD-1 and CD57 was examined by flow cytometry in the respective T cell subpopulations.

Supplementary Figure 2 The histograms of geomean fluorescence intensity for inhibitory receptors in T cell subsets.

Supplementary Figure 3 The histograms of geomean fluorescence intensity for inhibitory receptors in T cell subsets included in the predictive model 2.

Supplementary Figure 4 Variable selection of laboratory examination items using LASSO regression. **(A)** The coefficient screening trajectory of all candidate variables associated with AF recurrence. **(B)** The association between log-transformed λ and partial likelihood deviance for AF recurrence. The red dots with gray error bars represent the mean partial likelihood deviance and 95% CI. The left dashed line denotes the optimal λ at minimum deviance, and the right dashed line denotes the λ value selected by the one standard error rule.

Supplementary Figure 5 Variable selection of data related to exhausted T cells using LASSO regression. **(A)** The coefficient screening trajectory of all candidate variables associated with AF recurrence. **(B)** The association between log-transformed λ and partial likelihood deviance for AF recurrence. The red dots with gray error bars represent the mean partial likelihood deviance and 95% CI. The left dashed line denotes the optimal λ at minimum deviance, and the right dashed line denotes the λ value selected by the one standard error rule.
