# Supplementary figures and images for: Association between T cell exhaustion and the recurrence of atrial fibrillation after cryoballoon ablation
Source: Front Immunol. 2026 Apr 14;17:1767253. doi: 10.3389/fimmu.2026.1767253 (PMC13121131; doi:10.3389/fimmu.2026.1767253)

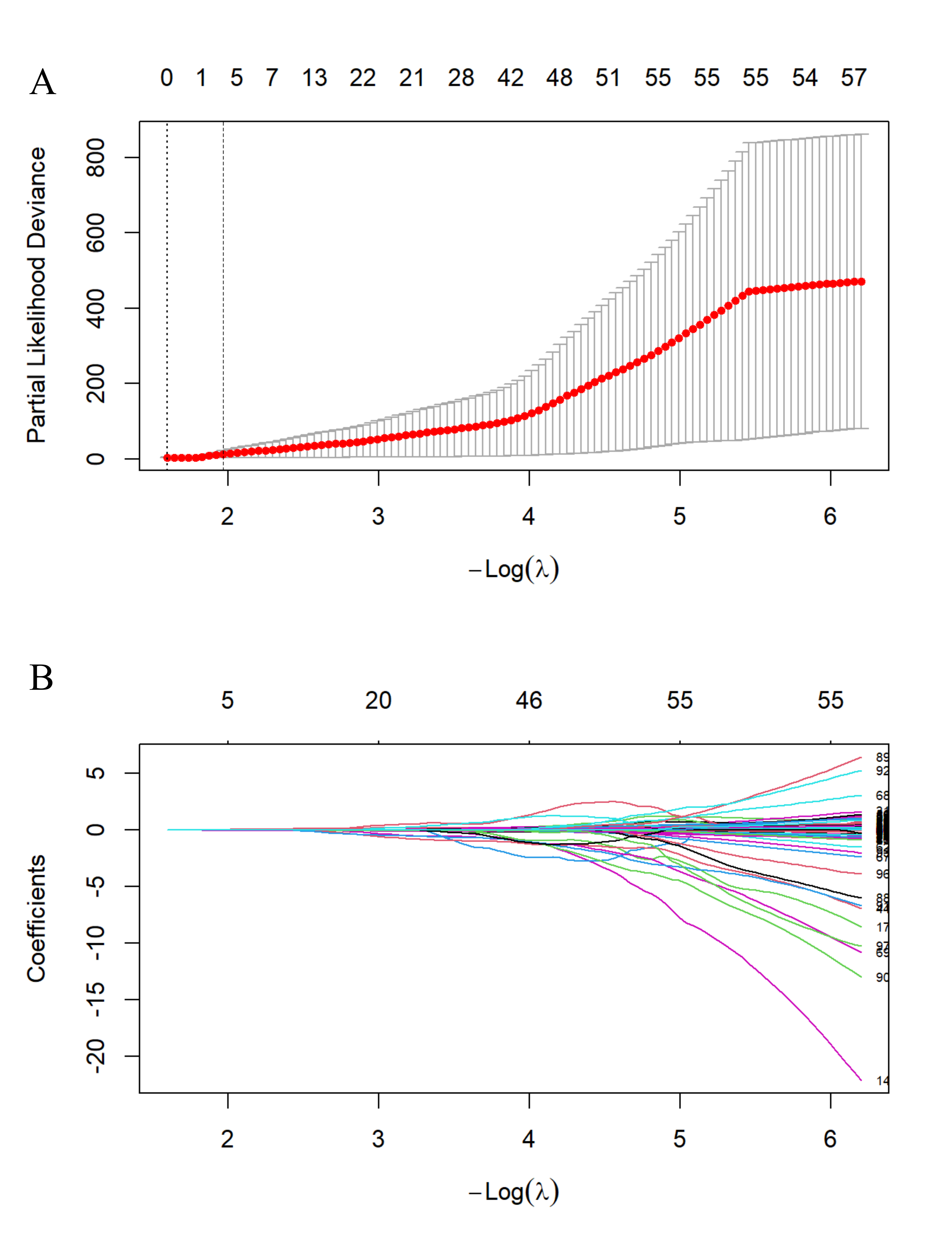

Supplement: Supplementary Figure 3 — The histograms of geomean fluorescence intensity for inhibitory receptors in T cell subsets included in the predictive model 2. [file Image3.tif]

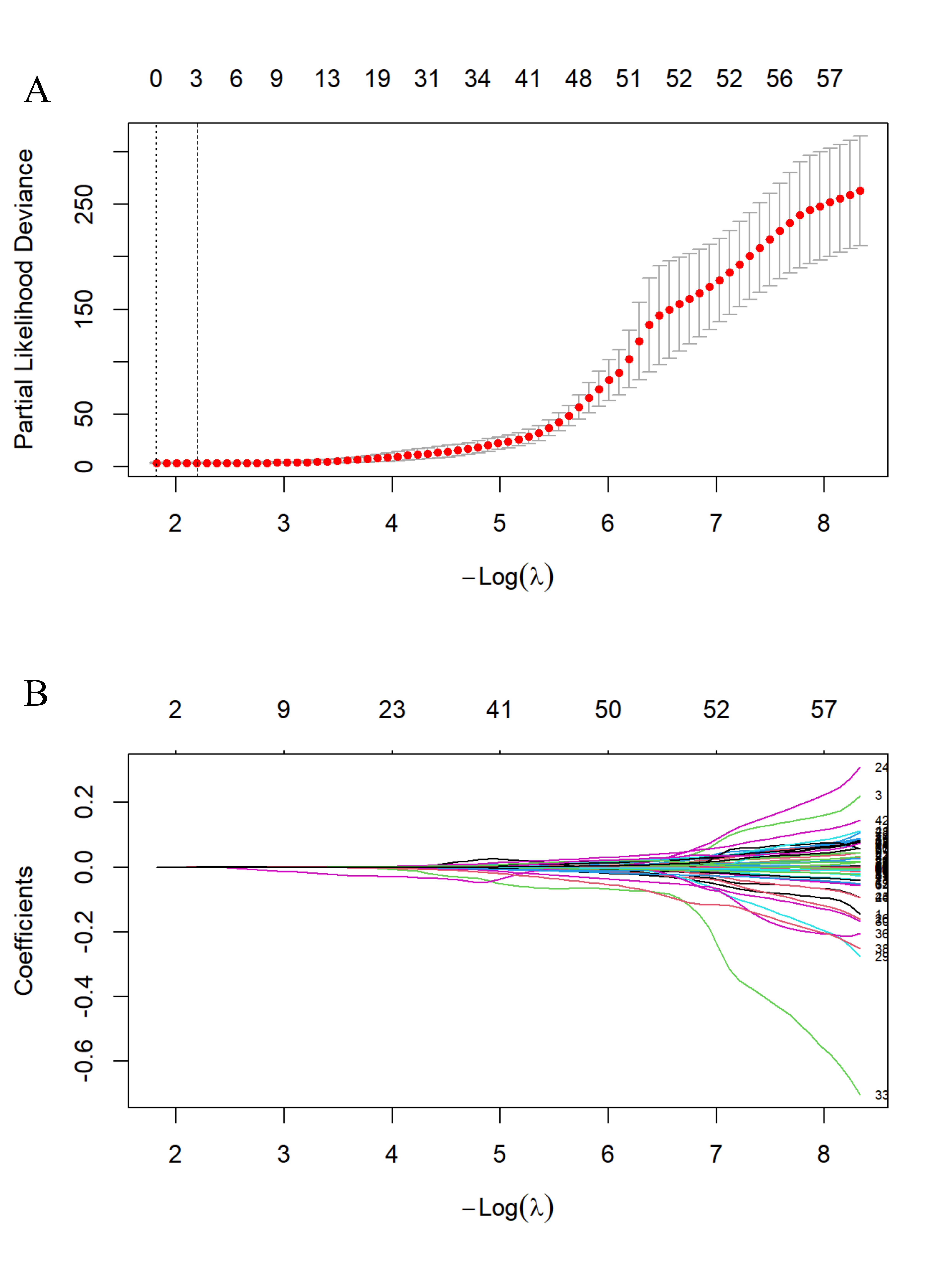

Supplement: Supplementary Figure 4 — Variable selection of laboratory examination items using LASSO regression. (A) The coefficient screening trajectory of all candidate variables associated with AF recurrence. (B) The association between log-transformed λ and partial likelihood deviance for AF recurrence. The red dots with gray error bars represent the mean partial likelihood deviance and 95% CI. The left dashed line denotes the optimal λ at minimum deviance, and the right dashed line denotes the λ value selected by the one standard error rule. [file Image4.tif]

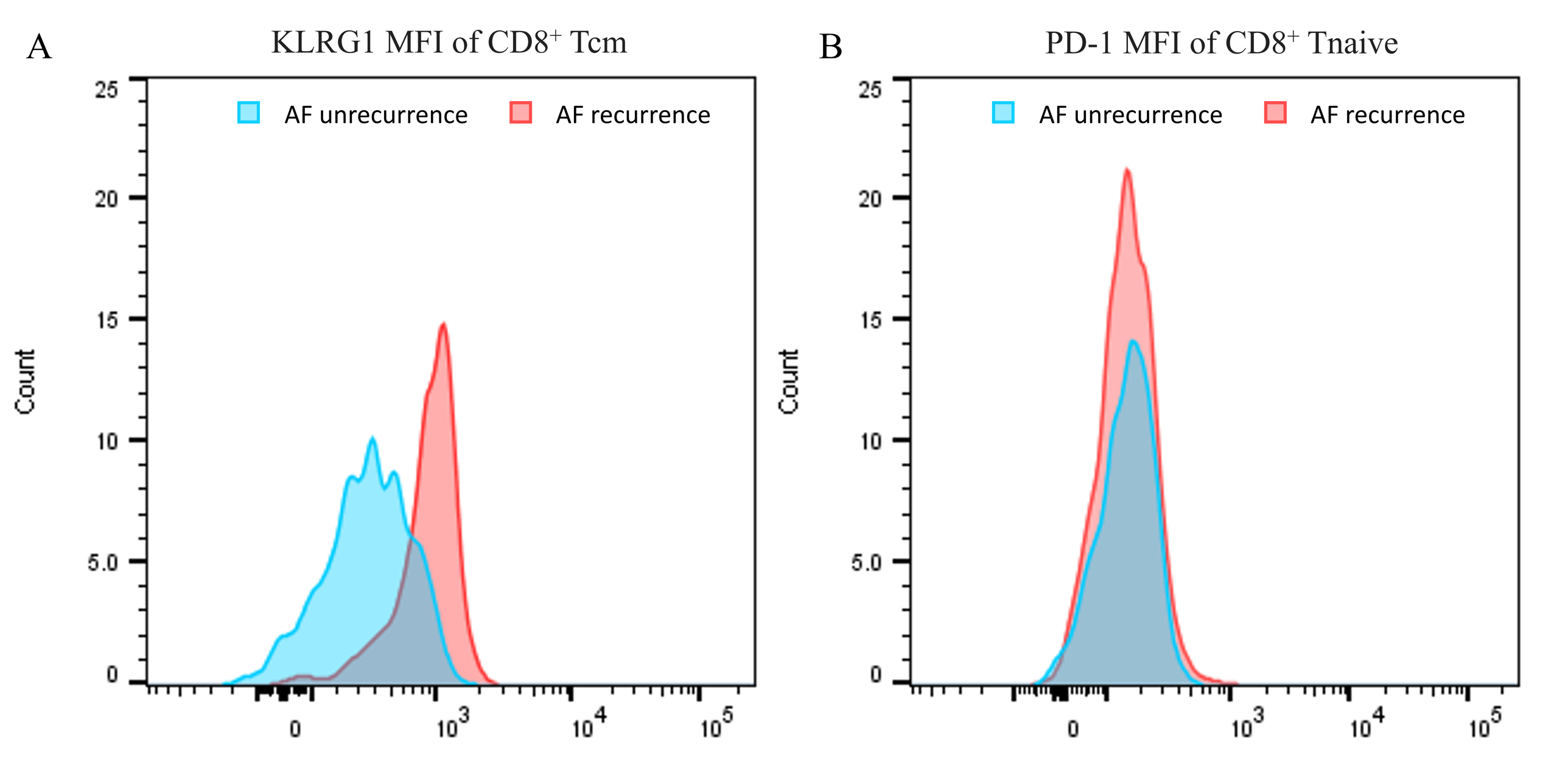

Supplement: Supplementary Figure 5 — Variable selection of data related to exhausted T cells using LASSO regression. (A) The coefficient screening trajectory of all candidate variables associated with AF recurrence. (B) The association between log-transformed λ and partial likelihood deviance for AF recurrence. The red dots with gray error bars represent the mean partial likelihood deviance and 95% CI. The left dashed line denotes the optimal λ at minimum deviance, and the right dashed line denotes the λ value selected by the one standard error rule. [file Image5.tif]
