# Supplementary material for: Association between T cell exhaustion and the recurrence of atrial fibrillation after cryoballoon ablation
Source: Front Immunol. 2026 Apr 14;17:1767253. doi: 10.3389/fimmu.2026.1767253 (PMC13121131; doi:10.3389/fimmu.2026.1767253)
Supplement: Supplementary file 6 [file Table1.docx]

**Table S1 The gating strategy for the identification of T cell subsets and inhibitory receptors**

| Antibodys | Clone | Concentration | Supplier | Product code |
| --- | --- | --- | --- | --- |
| APC anti-human CD3 | HIT3a | 1:25 | Biolegend | 300312 |
| APC-CY7 anti-human CD4 | SK3 | 1:25 | Biolegend | 344615 |
| AF700 anti-human CD8 | [SK1](https://www.biolegend.com/en-us/search-results?Clone=SK1) | 1:25 | Biolegend | 344723 |
| BV421 anti-human CD197 | G043H7 | 1:50 | Biolegend | 353207 |
| PE-CY7 anti-human CD28 | CD28.2 | 1:50 | Biolegend | 302925 |
| FITC anti-human CD57 | HNK-1 | 1:50 | Biolegend | 359603 |
| PE anti-human CD95 | DX2 | 1:50 | Biolegend | 305607 |
| BV 510 anti-human CD45RA | HI100 | 1:50 | Biolegend | 304141 |
| BV 650 anti-human CD27 | O323 | 1:50 | Biolegend | 302827 |
| BV 510 anti-human PD-1 | NAT105 | 1:50 | Biolegend | 367423 |
| PerCP-CY5.5 anti-human KLRG1 | SA231A2 | 1:50 | Biolegend | 367707 |
| PE-CY5 anti-human Tim-3 | F38-2E2 | 1:50 | Biolegend | 345051 |
| PE-Dazzle 594 anti-human LAG-3 | [11C3C65](https://www.biolegend.com/en-us/search-results?Clone=11C3C65) | 1:50 | Biolegend | 369331 |
