# Supplementary material for: Association between T cell exhaustion and the recurrence of atrial fibrillation after cryoballoon ablation
Source: Front Immunol. 2026 Apr 14;17:1767253. doi: 10.3389/fimmu.2026.1767253 (PMC13121131; doi:10.3389/fimmu.2026.1767253)
Supplement: Supplementary file 7 [file Table2.docx]

**Table S2 Flow cytometry results in the study population**

| **Variable** | **All patients** | **AF unrecurrence Group** | **AF recurrence Group** | **p** |
| --- | --- | --- | --- | --- |
|  | N=85 | N=50 | N=35 |  |
|  |  |  |  |  |
| KLRG1 of CD3^+^ T cell (MFI) | 532 [471;616] | 522 [438;593] | 579 [493;628] | 0.089 |
| KLRG1 of CD4^+^ T cell (MFI) | 396 [336;446] | 386 [324;436] | 407 [360;450] | 0.166 |
| KLRG1 of CD4^+^ Tcm cell (MFI) | 446 [364;474] | 434 [360;472] | 450 [374;484] | 0.469 |
| KLRG1 of CD4^+^ Tem cell (MFI) | 444 [349;516] | 429 [332;510] | 460 [410;548] | 0.022 |
| KLRG1 of CD4^+^ Temra cell (MFI) | 452 [296;659] | 398 [278;596] | 578 [398;700] | 0.012 |
| KLRG1 of CD4^+^ Tnaive cell (MFI) | 278 [249;304] | 276 [244;297] | 283 [264;306] | 0.284 |
| KLRG1 of CD4^+^ Tscm cell (MFI) | 439 [395;482] | 432 [390;466] | 451 [418;487] | 0.130 |
| KLRG1 of CD8^+^ T cell (MFI) | 824 ± 189 | 794 ± 184 | 867 ± 191 | 0.081 |
| KLRG1 of CD8^+^ Tcm cell (MFI) | 797 [705;897] | 756 [702;852] | 829 [718;946] | 0.066 |
| KLRG1 of CD8^+^ Tem cell (MFI) | 777 ± 202 | 744 ± 192 | 825 ± 209 | 0.073 |
| KLRG1 of CD8^+^ Temra cell (MFI) | 882 ± 208 | 854 ± 212 | 923 ± 199 | 0.131 |
| KLRG1 of CD8^+^ Tnaive cell (MFI) | 578 [491;646] | 558 [490;620] | 620 [502;688] | 0.043 |
| KLRG1 of CD8^+^ Tscm cell (MFI) | 926 [823;1093] | 922 [804;1029] | 926 [882;1118] | 0.180 |
| LAG-3 of CD3^+^ T cell (MFI) | 627 ± 172 | 621 ± 181 | 636 ± 160 | 0.678 |
| LAG-3 of CD4^+^ T cell (MFI) | 673 [600;809] | 678 [599;804] | 668 [604;819] | 0.986 |
| LAG-3 of CD4^+^ Tcm cell (MFI) | 1061 [898;1307] | 1082 [893;1328] | 1046 [916;1291] | 0.658 |
| LAG-3 of CD4^+^ Tem cell (MFI) | 790 [675;1094] | 791 [688;1067] | 780 [670;1144] | 0.989 |
| LAG-3 of CD4^+^ Temra cell (MFI) | 500 [367;634] | 463 [318;654] | 529 [413;628] | 0.192 |
| LAG-3 of CD4^+^ Tnaive cell (MFI) | 338 [290;378] | 324 [287;364] | 356 [292;381] | 0.198 |
| LAG-3 of CD4^+^ Tscm cell (MFI) | 911 [840;960] | 907 [829;976] | 911 [854;958] | 0.979 |
| LAG-3 of CD8^+^ T cell (MFI) | 583 ± 167 | 574 ± 172 | 597 ± 161 | 0.534 |
| LAG-3 of CD8^+^ Tcm cell (MFI) | 937 [810;1195] | 946 [825;1190] | 912 [798;1210] | 0.464 |
| LAG-3 of CD8^+^ Tem cell (MFI) | 569 [455;796] | 582 [460;802] | 539 [454;790] | 0.652 |
| LAG-3 of CD8^+^ Temra cell (MFI) | 502 [436;625] | 488 [428;620] | 517 [454;636] | 0.389 |
| LAG-3 of CD8^+^ Tnaive cell (MFI) | 384 [335;457] | 378 [318;447] | 398 [351;458] | 0.208 |
| LAG-3 of CD8^+^ Tscm cell (MFI) | 1074 ± 164 | 1064 ± 160 | 1088 ± 171 | 0.529 |
| TIM-3 of CD3^+^ T cell (MFI) | 3947 ± 928 | 3876 ± 971 | 4050 ± 866 | 0.389 |
| TIM-3 of CD4^+^ T cell (MFI) | 4683 [3830;5450] | 4556 [3726;5510] | 4791 [3948;5246] | 0.526 |
| TIM-3 of CD4^+^ Tcm cell (MFI) | 4912 [4327;5561] | 4890 [4265;5571] | 5005 [4350;5375] | 0.886 |
| TIM-3 of CD4^+^ Tem cell (MFI) | 3789 ± 823 | 3741 ± 806 | 3858 ± 854 | 0.527 |
| TIM-3 of CD4^+^ Temra cell (MFI) | 4147 ± 993 | 4072 ± 974 | 4254 ± 1024 | 0.413 |
| TIM-3 of CD4^+^ Tnaive cell (MFI) | 5711 ± 934 | 5662 ± 946 | 5780 ± 927 | 0.570 |
| TIM-3 of CD4^+^ Tscm cell (MFI) | 5834 ± 838 | 5773 ± 802 | 5923 ± 892 | 0.429 |
| TIM-3 of CD8^+^ T cell (MFI) | 3463 ± 801 | 3399 ± 860 | 3555 ± 712 | 0.365 |
| TIM-3 of CD8^+^ Tcm cell (MFI) | 3789 ± 645 | 3746 ± 706 | 3850 ± 549 | 0.446 |
| TIM-3 of CD8^+^ Tem cell (MFI) | 3060 ± 785 | 3033 ± 844 | 3099 ± 703 | 0.695 |
| TIM-3 of CD8^+^ Temra cell (MFI) | 3468 ± 778 | 3404 ± 850 | 3559 ± 664 | 0.348 |
| TIM-3 of CD8^+^ Tnaive cell (MFI) | 3745 ± 693 | 3680 ± 736 | 3839 ± 626 | 0.288 |
| TIM-3 of CD8^+^ Tscm cell (MFI) | 4595 ± 726 | 4521 ± 798 | 4700 ± 605 | 0.243 |
| PD-1 of CD3^+^ T cell (MFI) | 240 [188;291] | 237 [166;282] | 254 [198;294] | 0.230 |
| PD-1 of CD4^+^ T cell (MFI) | 266 ± 99.2 | 254 ± 105 | 284 ± 89.0 | 0.155 |
| PD-1 of CD4^+^ Tcm cell (MFI) | 308 [261;348] | 308 [239;342] | 310 [267;358] | 0.414 |
| PD-1 of CD4^+^ Tem cell (MFI) | 176 ± 62.1 | 174 ± 66.0 | 180 ± 56.8 | 0.617 |
| PD-1 of CD4^+^ Temra cell (MFI) | 174 ± 56.5 | 169 ± 61.4 | 180 ± 48.9 | 0.385 |
| PD-1 of CD4^+^ Tnaive cell (MFI) | 332 [260;467] | 321 [252;464] | 362 [282;474] | 0.132 |
| PD-1 of CD4^+^ Tscm cell (MFI) | 360 [305;461] | 344 [285;447] | 378 [326;488] | 0.067 |
| PD-1 of CD8^+^ T cell (MFI) | 219 [170;288] | 218 [142;280] | 223 [178;288] | 0.523 |
| PD-1 of CD8^+^ Tcm cell (MFI) | 279 [217;313] | 280 [208;318] | 276 [232;308] | 0.529 |
| PD-1 of CD8^+^ Tem cell (MFI) | 143 ± 58.5 | 142 ± 62.2 | 144 ± 53.5 | 0.881 |
| PD-1 of CD8^+^ Temra cell (MFI) | 204 ± 80.6 | 195 ± 79.5 | 216 ± 81.7 | 0.243 |
| PD-1 of CD8^+^ Tnaive cell (MFI) | 367 [272;510] | 362 [266;495] | 419 [278;557] | 0.165 |
| PD-1 of CD8^+^ Tscm cell (MFI) | 325 [275;437] | 324 [261;408] | 345 [290;520] | 0.105 |
| CD28 of CD3^+^ T cell (MFI) | 1678 [1248;2318] | 1696 [1257;2208] | 1641 [1256;2556] | 0.535 |
| CD28 of CD4^+^ T cell (MFI) | 3032 [2451;3608] | 2821 [2387;3511] | 3076 [2510;3644] | 0.348 |
| CD28 of CD4^+^ Tcm cell (MFI) | 4236 [3468;4895] | 4270 [3194;4992] | 4002 [3644;4796] | 0.796 |
| CD28 of CD4^+^ Tem cell (MFI) | 2478 [2037;3408] | 2444 [2058;3437] | 3091 [2029;3384] | 0.782 |
| CD28 of CD4^+^ Temra cell (MFI) | 1696 [1344;2394] | 1582 [1238;2241] | 1986 [1397;2598] | 0.146 |
| CD28 of CD4^+^ Tnaive cell (MFI) | 2837 [2408;3235] | 2811 [2259;3226] | 2854 [2564;3192] | 0.377 |
| CD28 of CD4^+^ Tscm cell (MFI) | 3637 [3226;4118] | 3616 [3131;4120] | 3706 [3350;4046] | 0.478 |
| CD28 of CD8^+^ T cell (MFI) | 1050 [753;1293] | 1052 [750;1291] | 978 [781;1287] | 0.886 |
| CD28 of CD8^+^ Tcm cell (MFI) | 2453 ± 817 | 2535 ± 859 | 2335 ± 750 | 0.256 |
| CD28 of CD8^+^ Tem cell (MFI) | 1211 [830;1698] | 1320 [874;1702] | 1106 [822;1693] | 0.406 |
| CD28 of CD8^+^ Temra cell (MFI) | 736 [582;919] | 776 [569;896] | 715 [611;930] | 0.605 |
| CD28 of CD8^+^ Tnaive cell (MFI) | 1446 [1110;1777] | 1446 [1080;1790] | 1446 [1206;1739] | 0.929 |
| CD28 of CD8^+^ Tscm cell (MFI) | 2130 [1761;2740] | 2253 [1806;2831] | 2072 [1704;2531] | 0.210 |
| CD57 of CD3^+^ T cell (MFI) | 71.0 [52.6;100] | 71.2 [48.1;102] | 70.0 [56.5;87.6] | 0.858 |
| CD57 of CD4^+^ T cell (MFI) | 30.9 [23.8;42.9] | 30.0 [23.1;41.1] | 33.2 [25.7;45.5] | 0.262 |
| CD57 of CD4^+^ Tcm cell (MFI) | 23.4 [19.4;26.4] | 22.6 [20.1;26.3] | 23.6 [19.4;26.8] | 0.623 |
| CD57 of CD4^+^ Tem cell (MFI) | 40.1 [24.3;73.9] | 32.2 [21.9;61.4] | 49.4 [32.1;81.5] | 0.055 |
| CD57 of CD4^+^ Temra cell (MFI) | 53.9 [22.1;290] | 38.0 [20.1;414] | 105 [26.8;244] | 0.324 |
| CD57 of CD4^+^ Tnaive cell (MFI) | 19.0 [15.0;22.5] | 18.9 [14.8;24.4] | 19.4 [15.1;21.4] | 0.929 |
| CD57 of CD4^+^ Tscm cell (MFI) | 35.7 [30.6;43.1] | 35.5 [30.5;41.7] | 36.0 [30.9;47.6] | 0.562 |
| CD57 of CD8^+^ T cell (MFI) | 175 [92.6;283] | 166 [84.9;286] | 195 [117;281] | 0.259 |
| CD57 of CD8^+^ Tcm cell (MFI) | 42.8 [31.3;60.3] | 40.5 [29.0;57.1] | 44.5 [36.2;74.9] | 0.082 |
| CD57 of CD8^+^ Tem cell (MFI) | 88.7 [48.6;154] | 76.7 [37.6;120] | 100 [73.6;168] | 0.012 |
| CD57 of CD8^+^ Temra cell (MFI) | 309 [175;609] | 290 [174;618] | 369 [212;560] | 0.592 |
| CD57 of CD8^+^ Tnaive cell (MFI) | 55.8 [40.8;78.3] | 54.4 [38.1;72.2] | 64.0 [44.0;86.7] | 0.168 |
| CD57 of CD8^+^ Tscm cell (MFI) | 77.6 [58.8;136] | 72.1 [56.1;122] | 84.2 [70.8;140] | 0.065 |
| CD27 of CD3^+^ T cell (MFI) | 975 ± 344 | 969 ± 358 | 983 ± 328 | 0.853 |
| CD27 of CD4^+^ T cell (MFI) | 1029 ± 344 | 1032 ± 362 | 1024 ± 323 | 0.909 |
| CD27 of CD4^+^ Tcm cell (MFI) | 1280 ± 309 | 1293 ± 279 | 1261 ± 350 | 0.651 |
| CD27 of CD4^+^ Tem cell (MFI) | 539 ± 140 | 546 ± 134 | 528 ± 151 | 0.571 |
| CD27 of CD4^+^ Temra cell (MFI) | 743 ± 182 | 756 ± 189 | 725 ± 173 | 0.435 |
| CD27 of CD4^+^ Tnaive cell (MFI) | 1830 [1588;2185] | 1824 [1553;2177] | 1860 [1634;2178] | 0.908 |
| CD27 of CD4^+^ Tscm cell (MFI) | 1844 [1556;2159] | 1816 [1563;2152] | 1864 [1552;2191] | 0.614 |
| CD27 of CD8^+^ T cell (MFI) | 957 [727;1152] | 1003 [716;1153] | 941 [798;1135] | 0.932 |
| CD27 of CD8^+^ Tcm cell (MFI) | 1095 ± 294 | 1098 ± 283 | 1091 ± 313 | 0.917 |
| CD27 of CD8^+^ Tem cell (MFI) | 556 ± 191 | 565 ± 206 | 543 ± 169 | 0.590 |
| CD27 of CD8^+^ Temra cell (MFI) | 743 ± 182 | 756 ± 189 | 725 ± 173 | 0.435 |
| CD27 of CD8^+^ Tnaive cell (MFI) | 1862 [1542;2295] | 1846 [1524;2244] | 1933 [1691;2311] | 0.386 |
| CD27 of CD8^+^ Tscm cell (MFI) | 1596 [1350;1840] | 1614 [1382;1776] | 1578 [1295;1870] | 0.652 |
| (CD4^+^ Tcm cell) / (CD4^+^ T cell)(%) | 0.30 ± 0.10 | 0.30 ± 0.12 | 0.30 ± 0.07 | 0.989 |
| (CD4^+^ Tem cell) / (CD4^+^ T cell)(%) | 0.34 [0.27;0.48] | 0.34 [0.27;0.51] | 0.34 [0.26;0.44] | 0.758 |
| (CD4^+^ Temra cell) / (CD4^+^ T cell)(%) | 0.03 [0.01;0.06] | 0.04 [0.02;0.08] | 0.02 [0.01;0.05] | 0.084 |
| (CD4^+^ Tnaive cell) / (CD4^+^ T cell)(%) | 0.22 [0.14;0.30] | 0.17 [0.13;0.28] | 0.26 [0.20;0.31] | 0.039 |
| (CD4^+^ Tscm cell) / (CD4^+^ T cell)(%) | 0.04 [0.02;0.07] | 0.04 [0.02;0.08] | 0.04 [0.03;0.07] | 0.794 |
| (CD8^+^ Tcm cell) / (CD8^+^ T cell)(%) | 0.07 [0.05;0.12] | 0.08 [0.05;0.12] | 0.07 [0.06;0.12] | 0.710 |
| (CD8^+^ Tem cell) / (CD8^+^ T cell)(%) | 0.26 [0.18;0.36] | 0.24 [0.16;0.36] | 0.26 [0.23;0.35] | 0.401 |
| (CD8^+^ Temra cell) / (CD8^+^ T cell)(%) | 0.49 ± 0.18 | 0.50 ± 0.18 | 0.49 ± 0.18 | 0.865 |
| (CD8^+^ Tnaive cell) / (CD8^+^ T cell)(%) | 0.08 [0.05;0.14] | 0.08 [0.05;0.14] | 0.07 [0.04;0.13] | 0.747 |
| (CD8^+^ Tscm cell) / (CD8^+^ T cell)(%) | 0.03 [0.02;0.05] | 0.03 [0.02;0.06] | 0.03 [0.02;0.04] | 0.356 |
| (CD4^+^ Tcm cell) / (CD3^+^ T cell)(%) | 0.14 [0.11;0.19] | 0.14 [0.11;0.19] | 0.15 [0.12;0.17] | 0.737 |
| (CD4^+^ Tem cell) / (CD3^+^ T cell)(%) | 0.17 [0.13;0.22] | 0.17 [0.13;0.21] | 0.16 [0.13;0.24] | 0.761 |
| (CD4^+^ Temra cell) / (CD3^+^ T cell)(%) | 0.02 [0.01;0.03] | 0.02 [0.01;0.03] | 0.02 [0.00;0.03] | 0.209 |
| (CD4^+^ Tnaive cell) / (CD3^+^ T cell)(%) | 0.10 [0.06;0.17] | 0.08 [0.06;0.16] | 0.12 [0.07;0.18] | 0.092 |
| (CD4^+^ Tscm cell) / (CD3^+^ T cell)(%) | 0.02 [0.01;0.04] | 0.02 [0.01;0.04] | 0.02 [0.01;0.04] | 0.949 |
| (CD8^+^ Tcm cell) / (CD3^+^ T cell)(%) | 0.03 [0.02;0.04] | 0.02 [0.02;0.04] | 0.03 [0.02;0.05] | 0.125 |
| (CD8^+^ Tem cell) / (CD3^+^ T cell)(%) | 0.09 [0.06;0.12] | 0.08 [0.05;0.12] | 0.10 [0.07;0.14] | 0.133 |
| (CD8^+^ Temra cell) / (CD3^+^ T cell)(%) | 0.19 ± 0.10 | 0.19 ± 0.11 | 0.19 ± 0.09 | 0.947 |
| (CD8^+^ Tnaive cell) / (CD3^+^ T cell)(%) | 0.03 [0.02;0.04] | 0.03 [0.02;0.04] | 0.03 [0.01;0.04] | 0.989 |
| (CD8^+^ Tscm cell) / (CD3^+^ T cell)(%) | 0.30 ± 0.10 | 0.30 ± 0.12 | 0.30 ± 0.07 | 0.937 |
| CD4^+^ Tcm count (/ml) | 274550 [143543;428238] | 253518 [125393;408099] | 298907 [217782;445984] | 0.161 |
| CD4^+^ Tem count (/ml) | 306803 [179038;458432] | 309500 [174242;398638] | 306803 [210904;466995] | 0.448 |
| CD4^+^ Temra count (/ml) | 24646 [11884;48018] | 28044 [19114;45846] | 23232 [9045;50578] | 0.239 |
| CD4^+^ Tnaive count (/ml) | 227888 [89752;338613] | 163097 [80087;266035] | 261539 [137687;354281] | 0.016 |
| CD4^+^ Tscm count (/ml) | 42794 [15626;86333] | 32783 [11795;89250] | 51541 [23166;71372] | 0.211 |
| CD8^+^ Tcm count (/ml) | 46207 [24694;84480] | 42714 [23505;67223] | 51368 [28503;118570] | 0.138 |
| CD8^+^ Tem count (/ml) | 154386 [78024;273637] | 142059 [72302;253400] | 168333 [91308;365788] | 0.246 |
| CD8^+^ Temra count (/ml) | 293170 [169816;586794] | 276337 [156952;497364] | 371654 [184548;721930] | 0.215 |
| CD8^+^ Tnaive count (/ml) | 42908 [24876;87608] | 37938 [26180;72526] | 50403 [24422;151782] | 0.313 |
| CD8^+^ Tscm count (/ml) | 17329 [12122;38147] | 16712 [10152;37111] | 18740 [13608;39310] | 0.459 |
